# Dl-3-n-butylphthalide attenuates cerebral ischemia/reperfusion injury in mice through AMPK-mediated mitochondrial fusion

**DOI:** 10.3389/fphar.2024.1357953

**Published:** 2024-02-22

**Authors:** Ting Zhu, Shanshan Dong, Na Qin, Rujuan Liu, Liuliu Shi, Qi Wan

**Affiliations:** ^1^ Department of Pathophysiology, Institute of Neuroregeneration & Neurorehabilitation, School of Basic Medicine, Qingdao University, Qingdao, China; ^2^ Department of Rehabilitation Medicine, The Affiliated Hospital of Qingdao University, Qingdao, China; ^3^ Department of Neurosurgery, The Affiliated Hospital of Qingdao University, Qingdao, China

**Keywords:** ischemic stroke, Dl-3-n-butylphthalide, mitochondrial fusion, AMPK, Mfn1

## Abstract

**Introduction:** NBP is a compound isolated from celery seeds, which was approved by the National Medical Products Administration in 2002 for clinical treatment of ischemic stroke. However, in brain ischemia/reperfusion (I/R) injury, the related research on mitochondrial dynamics and its mechanism of action of NBP still need to be further studied. The aim of this study was to assess NBP on cerebral pathology in ischemic stroke *in vivo*, with a specific focus on the molecular mechanisms of how NBP promotes mitochondrial fusion.

**Methods:** Male C57BL/6 mice were utilized in this study and were subjected to middle cerebral artery occlusion/reperfusion (MCAO/R). Pre-ischemia, NBP was administered through intraperitoneal (i.p.) injection for 7 days.

**Results:** Our findings demonstrated that NBP effectively reduced infarct volume, improved neurological dysfunction, enhanced cerebral blood flow, and promoted mitochondrial fusion in mice subjected to MCAO/R. More importantly, the pro-fusion effects of NBP were found to be linked to the activation of AMPK/Mfn1 pathway, and with the activation of neurological function, which was partially eliminated by inhibitors of AMPK.

**Discussion:** Our results revealed that NBP is a novel mitochondrial fusion promoter in protecting against ischemic stroke through the AMPK-mediated Mfn1. These findings contribute to the understanding of novel mechanisms involved in the protection of neurological function following NBP treatment for ischemic stroke.

## Introduction

Stroke, a disease characterized by a substantial disability rate and elevated mortality rate, has emerged as a prominent contributor to global mortality rates in recent times, with ischemic stroke (IS) being the prevailing subtype (Wang et al., 2021; Xie et al., 2024). According to the latest epidemiological survey results, there are about 2.4 million new stroke patients in China every year, most of whom will be left with varying degrees of neurological dysfunction, which brings a serious burden to patients, families and society (Zhu et al., 2024). In recent years, extensive research has been conducted on the pathological mechanisms underlying brain ischemia/reperfusion (I/R) injury, including the excessive production of reactive oxygen species (ROS), disturbances in calcium ion (Ca^2+^) homeostasis, excitotoxic effects, apoptosis, and mitophagy. Notably, Ca^2+^ overload and ROS burst manifest in the initial phase of neuronal injury, leading to various modes of cell death, such as apoptosis and autophagy. The management of cerebral I/R injury continues to pose significant clinical difficulties, necessitating the identification of precise therapeutic targets for improved treatment outcomes and prognoses.

The dynamics of mitochondrial fusion and fission have a significant correlation with the onset and progression of cerebral I/R injury. While inhibiting excessive mitochondrial fission has proven advantageous for various diseases, it can have detrimental effects on the integrity of mitochondrial DNA (mtDNA) and respiratory complexes during cerebral I/R injury (Yang et al., 2021). Concurrently, mitochondrial fusion plays a crucial role in maintaining mitochondrial health. This process involves several proteins, including three membrane GTPases: mitofusin 1 (Mfn1), mitofusin 2 (Mfn2), and optic atrophy protein 1 (Opa1) (Wu et al., 2021). The process of mitochondrial fusion serves to enhance oxidative phosphorylation and facilitate the redistribution of mitochondrial DNA within both impaired and intact mitochondria (Huang et al., 2023). By increasing the expression of fusion-related proteins, the promotion of mitochondrial fusion effectively mitigates cerebral ischemic injury without compromising overall mitochondrial quantity in the basal brain. Consequently, this approach proves to be a safer alternative to inhibiting mitochondrial fission. Therefore, the identification and development of efficacious therapeutic medications that target the promotion of mitochondrial fusion hold substantial importance in the realm of ischemic stroke treatment.

NBP, a compound derived from celery seeds, was officially sanctioned by the National Medical Products Administration in 2002 for the therapeutic management of ischemic stroke (Wang S. et al., 2018). Extensive research has demonstrated the potential of NBP to enhance neurological recuperation in individuals suffering from acute stroke-induced disability (Wang S. et al., 2018), vascular dementia (Huang et al., 2017) and Alzheimer’s disease (Wang et al., 2019). The protective effects of NBP in ischemic stroke are believed to be attributed to its ability to ameliorate local microcirculation, suppress neuronal apoptosis, and mitigate neuroinflammation (Zhang et al., 2018; Wei et al., 2021). Recent studies have shown that NBP significantly alleviates demyelinating in white matter lesion models by promoting mitochondrial dynamics and improves spatial learning and memory in mice (Feng et al., 2021). However, in cerebral I/R injury, the related research on mitochondrial dynamics and its mechanism of action of NBP still need to be further studied. We investigated the effects of NBP on cerebral pathology in ischemic stroke *in vivo*, focusing on the molecular mechanisms by which NBP promotes mitochondrial fusion. Our results have revealed that NBP is a novel mitochondrial fusion promoter in protecting against ischemic stroke through the AMPK-mediated Mfn1.

## Methods

### Animals

All animal care and experimental procedures were approved by the Institutional Animal Care guidelines and the Animal Care and Ethics Committee of Qingdao University. C57BL/6 male mice (approval codes: QDU-AEC-2021101) weighing 20–25 g were purchased from Jinan Pengyue Experimental Animals Breeding Co., Ltd., were housed with a 12-hour light/dark cycle, with free access to food and water, at a temperature of 20°C–25°C and humidity of 50%–60%. The mice were given at least 3 days to get used to the new environment before being used in experiments. Every effort was made to minimize the number of mice used and minimize suffering.

### MCAO surgery

Following a 7-day injection period with either 20 mg kg^−1^ NBP or 0.9% normal saline, C57BL/6 mice were subjected to anesthesia using 3.5% isoflurane, with maintenance at 1.5% isoflurane throughout the MCAO surgery. To ensure a stable body temperature of 37°C, a heating pad was utilized. Under sterile conditions, a midline incision was made to expose the right neck vessels, allowing for discrete isolation of the right common carotid artery (CCA), external carotid artery (ECA), and internal carotid artery (ICA). The monofilament suture should be carefully inserted from the lumen of the CCA into the ICA, extending approximately 9–10 mm beyond the bifurcation of the CCA to effectively occlude the origin of the MCA. Following this, the incision on the neck should be sutured, and the mouse should be placed in a nursing box maintained at a temperature of 37°C ± 0.5°C. After a period of 1.5 h of arterial occlusion, the filament should be withdrawn to allow for blood reperfusion. In the case of Sham-operated control mice, the same procedure should be followed, with the exception of filament insertion.

### *In vivo* drug treatment

NBP (obtained from CSPC NBP Pharmaceutical Co., Ltd) was dissolved in 0.9% normal saline prior to administration. All mice were randomly divided into three groups, as followed: Sham group, MCAO/R group, and NBP group. For drug administration, NBP (20 mg kg^−1^) or 0.9% saline was given every day to continuous intraperitoneal injection for 7 days prior to MCAO surgery. To further estimate the neuroprotective effect of NBP, 24 h after MCAO/R, mice were measured for cerebral blood flow, neurological deficits score, and grasp strength test, after which mice were sacrificed for TTC, Western blotting, Cresyl violet staining, and immunofluorescence.

### Cerebral blood flow

The laser speckle blood monitor manufactured by PERIMED in Sweden was employed for the purpose of conducting laser scatter imaging on the brains of mice. Following a 24-hour period of blood reperfusion, the mice were subjected to anesthesia. Subsequently, an incision was made in the center of the mice’s scalp to expose the skull, and the laser speckle imaging procedure was carried out using the imager positioned directly above the skull.

### Grip-strength test

A grip force meter (HUAYON, China) was used to assess grip strength of mice. At 24 h after reperfusion, the mice were tested for grip strength. Mice were held by their tails and placed on the device, the mice were then gently pulled backward until loss of grip. Every mouse was tested for three times and calculate the mean of three measurements for each animal.

### TTC staining

At 24 h post-reperfusion, the infarct volume following MCAO was quantified using a solution of 2,3,5-triphenyltetrazolium chloride (TTC). The brain tissue was uniformly sectioned into six coronal slices, which were then incubated with a 2% TTC solution at 37°C for 15 min in the absence of light. Subsequently, the slices were fixed in 4% paraformaldehyde (PFA) at 4°C overnight. The normal tissue exhibited a deep red stain, while the infarcted region appeared white. To determine the infarct volume, TTC-stained sections were captured using photography, and the resulting images were subjected to analysis using ImageJ software.

### Nissl staining

Frozen sections of brain tissue were fixed with 4% paraformaldehyde for 15 min and washed three times with PBS. Cresyl Violet Staining (Solarbio, China) was added to brain slices and incubated at 55°C for 1 h. Wash off the excess staining and differentiate by Nissl differentiation solution for a few seconds until the background is nearly colorless. Seal with glycerin jelly mounting medium and photographed with upright microscope (Nikon, Japan) and analyzed with ImageJ.

### TUNEL staining

One Step TUNEL Apoptosis Assay Kit purchased from Beyotime was used to detect apoptotic cells. First, frozen sections of brain tissue were fixed with 4% paraformaldehyde for 30 min. Then, PBS wash three times, 5 min each time. Block with 10% goat serum and 0.3% Triton X-100 for 1 h. The block buffer was discarded, and appropriate amount of TUNEL detection solution (TdT enzyme: fluorescent labeling solution = 1: 9) was added, and incubated at 37°C for 1 h without light. Discard the detection solution, wash three times with PBS, and seal the tablet with a sealing solution containing DAPI. The results were taken by fluorescence microscope and analyzed by ImageJ.

### Transmission electron microscope (TEM)

After 24 h of the reperfusion, 1-cubic mm of fresh brain tissue was taken and rapidly immersed into the Glutaraldehyde at 4°C for 2–4 h. The tissue was rinsed with 0.1 M phosphate buffer 3 times, 15 min each time. The tissue was fixed with 1% osmic acid at room temperature for 2 h, and washed with 0.1 M phosphate buffer 3 times, 15 min each time. After gradient dehydration, infiltration and embedding, the tissue was cut into ultrathin slices of 60–80 nm. The sections were stained with 2% uranium acetate and lead citrate for 15 min, and then the sections were dried overnight. Then the samples were observed under a TEM, and images were collected and analyzed.

### Western blotting

The cerebral ischemic cortex was homogenized in RIPA buffer. The homogenized protein was centrifuged at high speed, then the supernatant was extracted and the precipitation was discarded. Then the total protein concentration was determined with the BCA kit. 20 μg total protein samples from cortex were separated using SDS-PAGE and transferred to a PVDF membrane. The membranes were blocked using blocking solution (5% non-fat milk in TBST, pH 7.4) at room temperature for 60 min. Discard blocking solution and incubation with the primary antibody overnight at 4°C. Primary antibodies included: Opa1 (1:2000, Proteintech), Mfn1 (1: 2000, Proteintech), Mfn2 (1: 2000, Proteintech) and β-actin (1: 5000, Proteintech), AMPK (1:4000, Abclonal), pAMPK (1:5000, Abclonal), NDUFB8 (1:500, Abclonal), SDHB (1:500, Abclonal), UQCRC2 (1:500, Abclonal), MTCO2 (1:500, Abclonal), ATP5A1 (1:500, Abclonal). The following day, membranes were washed with TBST (10 min × 3) each time and subsequently incubated with secondary antibodies (1: 8000) for 2 h at room temperature. Membranes were then washed three times with TBST (10 min × 3). Protein expression was examined by using Chemiluminescent HRP Substrate (Millipore) and Cytiva. Densitometric values was quantified with ImageJ and were normalized with respect to β-actin immunoreactivity to correct for any loading and transfer differences among samples.

### Prediction of NBP targets and molecular docking

The SDF file of NBP was acquired from PubChem (https://pubchem.ncbi.nlm.nih.gov/compound/11092), and the PharmMapper Server (http://www.lilab-ecust.cn/pharmmapper/) was employed using the “All Targets” model to forecast the potential targets. The procedure was documented with a submission ID. Autodock Vina 1.1.2 was utilized to assess the binding affinity and binding sites between NBP and AMPK. The structures of NBP were obtained from the PubChem database, while the AMPK structure was obtained through the QuickPrep procedure.

### Drug affinity responsive target stability (DARTS)

The tissue lysates were diluted to achieve a uniform final volume. Subsequently, they were incubated with NBP at concentrations of 1, 2, 4, and 8 mg/mL for a duration of 1.5 h, while being gently agitated on a shaker. Following this, the samples were subjected to a reaction with protease at a concentration of 2 μg/mL in a reaction buffer for a period of 10 min. Subsequently, the loading buffer was introduced, and the p-AMPK protein was analyzed through Western blotting.

### Statistical analysis

Experimental data were collected from three independent experiments and are expressed as the means ± standard errors (SEMs). All analyses were statistically evaluated using SPSS17 software (IBM Corporation, New York, NY, USA). Group differences after significant ANOVAs were measured by *post hoc* Bonferroni test. A *p*-value of less than 0.05 was considered statistically significant.

## Results

### NBP reduced cerebral infarction volume, improved neurological dysfunction, and increased cerebral blood flow in MCAO/R-induced mice

**FIGURE 1:**
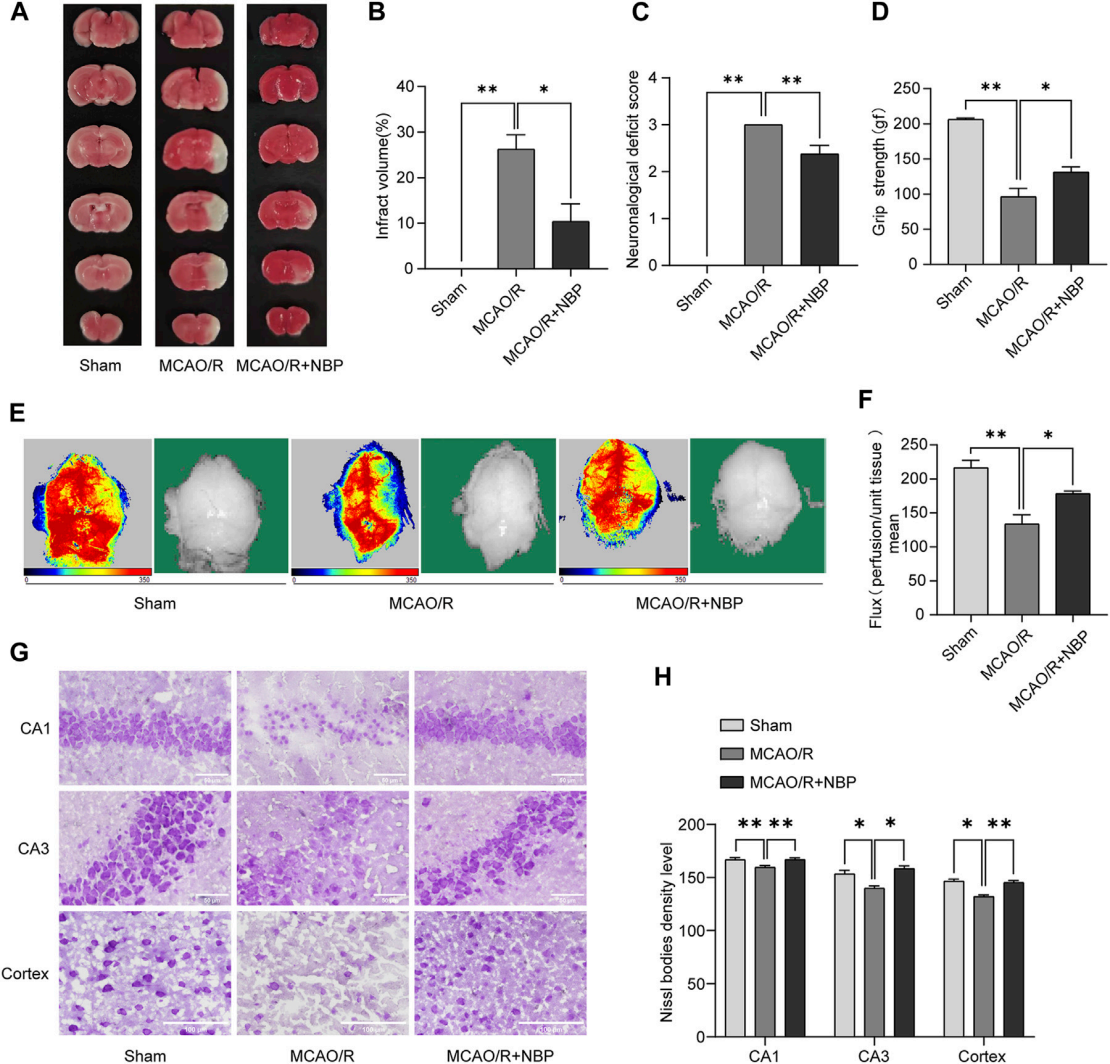
NBP alleviated infarction volume, neuronal loss, and facilitated restoration of cerebral blood flow in mice subjected to MCAO. NBP was intraperitoneally injected for 7 days before MCAO surgery. **(A)** Effects of NBP on infarction volumes. **(B)** Quantitative analysis of infarct volumes (*n* = 5). **(C)** Longa score in mice with MCAO/R at 24 h after reperfusion (*n* = 8). **(D)** Grasping ability test in mice with MCAO/R at 24 h after reperfusion (*n* = 6–9). **(E)** The images of cerebral blood flow of cortex. The magnitude of cerebral blood flow is represented by different colors, with blue to red indicating low to high. **(F)** Quantitative analysis of cerebral blood flow in different groups (*n* = 6). **(G)** Nissl staining of the hippocampal and cortex regions of each group (*n* = 3–4). **(H)** Nissl bodies density level in hippocampal CA1, CA3 and cortex regions in all the groups. Scale bar = 50 μm in hippocampal region, scale bar = 100 μm in cortex region. Data are expressed as the mean ± SEM and were analyzed by ANOVA, *p < 0.05 and **p < 0.01.

TTC staining and neurologic deficit test were conducted to evaluate the extent of infarct volume and neurological function 24 h post-reperfusion. As illustrated in Figures 1A, B, the mice subjected to MCAO/R treatment exhibited a distinct demarcation of infarction within the cerebral infarction core and penumbra. Notably, the administration of NBP significantly reduced the infarct volume in comparison to the MCAO/R group. Figures 1C, D also showed that treatment with NBP could improve the neurological deficits after I/R injury in mice.

Cerebral blood flow in mice was evaluated by Laser Doppler blood flow. As shown in Figures 1E, F, the groups pretreated with 20 mg kg^−1^ NBP for 7 days showed significantly increased cerebral blood flow compared to the MCAO/R group.

### NBP inhibited neuronal loss in MCAO/R-induced mice

As shown in Figures 1G, H, the Nissl staining analysis revealed that most neurons in the hippocampal CA1, CA3 and cortex regions in the MCAO/R group exhibited a shrunken phenotype, weak staining and were distributed irregularly. This observation indicated that neurons were predominantly deteriorated and that many Nissl bodies were lost in these neurons. Conversely, the administration of NBP for a duration of 7 days before MCAO surgery resulted in strong staining and a well-organized arrangement of neurons in the hippocampal CA1, CA3 and cortex regions, in comparison to the MCAO/R group.

### NBP promoted mitochondrial fusion in MCAO/R-induced mice

**FIGURE 2:**
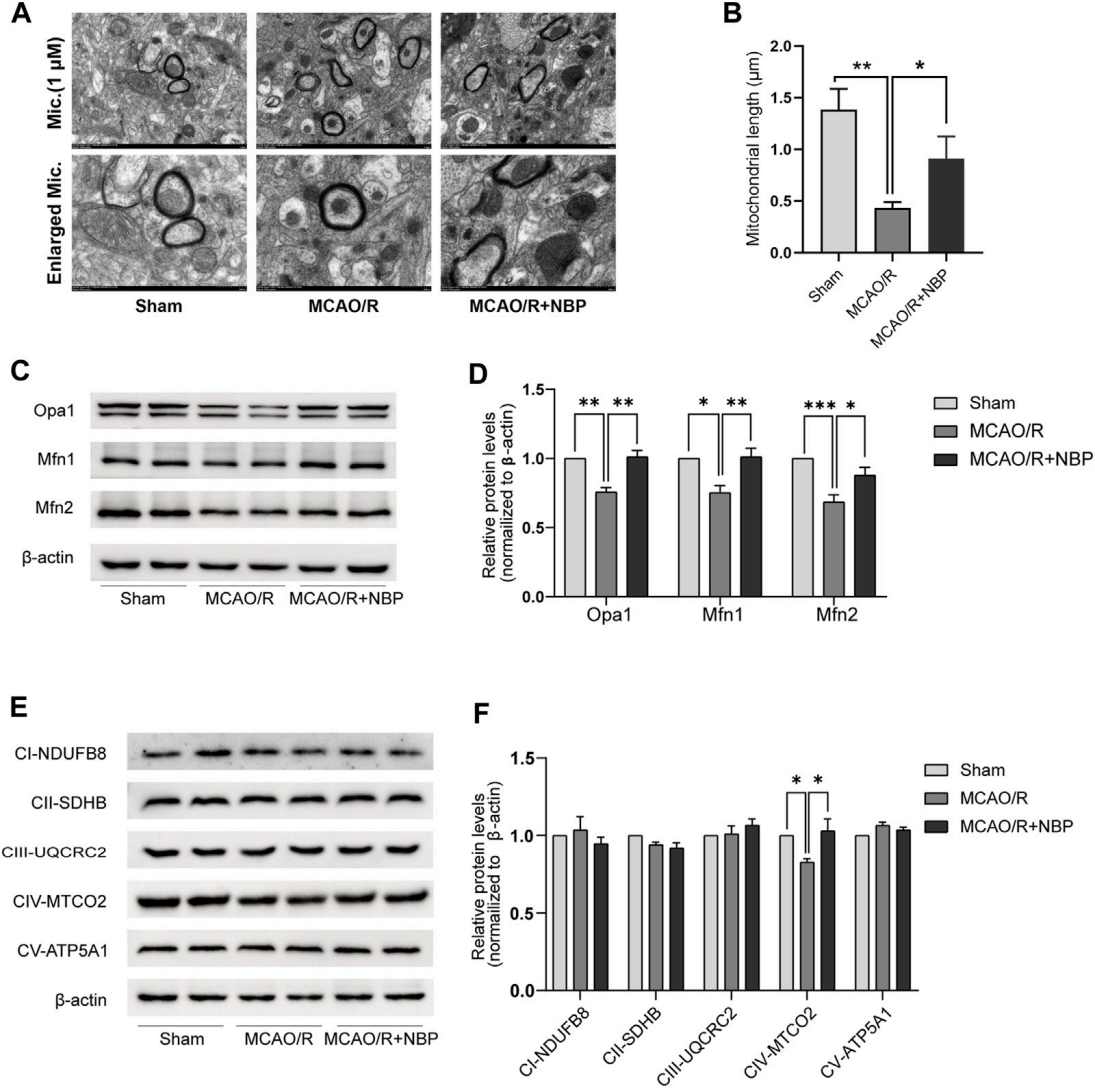
NBP promoted mitochondrial fusion and restored mitochondrial function in mice subjected to MCAO. **(A)** Mitochondrial morphology was observed by TEM. Scale bars = 500 nm. **(B)** Quantification of mean mitochondrial length. **(C, D)** Representative blots and quantitative analysis of mitochondrial fusion-related proteins including Opa1, Mfn1, and Mfn2 (*n* = 6). **(E, F)** Representative blots and quantitative analysis of mitochondrial respiratory chain complex I–V (CI–V) (*n* = 6). Data are expressed as the mean ± SEM and were analyzed by ANOVA, *p < 0.05, **p < 0.01 and ***p < 0.001.

The evaluation of mitochondrial fusion involved the determination of mitochondrial size. In comparison to the Sham group, the MCAO/R-induced brains exhibited a shorter mean mitochondrial length (Figure 2A). Notably, NBP pretreatment promoted mitochondrial fusion in MCAO/R-induced brains, as reflected by the increased mean mitochondrial length (Figure 2B).

Subsequently, an examination was conducted on the expression patterns of mitochondrial fission proteins (Opa1, Mfn1, and Mfn2) as well as mitochondrial respiratory chain complex I–V. It was observed that the levels of Opa1, Mfn1, and Mfn2, which are fusion proteins associated with mitochondria, were notably diminished in the tissues of mice induced with MCAO/R. However, the administration of NBP effectively reversed the decline in protein expression of Opa1, Mfn1, and Mfn2 in the brain tissues affected by MCAO/R, as depicted in Figures 2C, D. The expression of mitochondrial complexes IV was significantly diminished in the brain tissues induced by MCAO/R, among the mitochondrial respiratory chain complex I-V. However, this reduction was partially reversed by pretreatment with NBP, as evidenced by Figures 2E, F. Collectively, these findings suggest that NBP pretreatment enhances mitochondrial fusion and improves mitochondrial function in the context of cerebral I/R in mice.

### NBP activated AMPK following cerebral I/R injury

**FIGURE 3:**
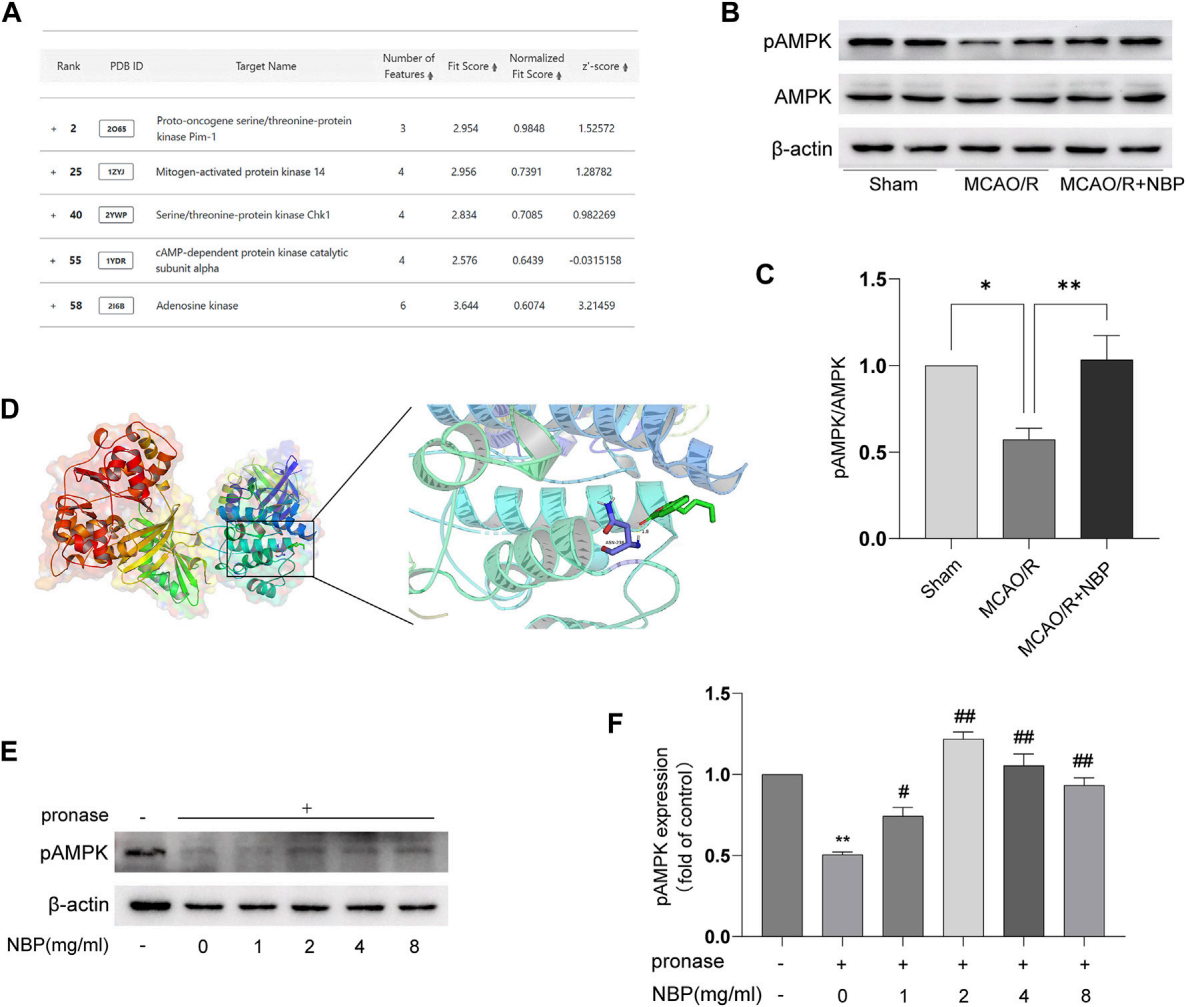
NBP directly binds to AMPK proteins. **(A)** Listing of potential candidates involved in kinase activity. Representative blots **(B)** and quantitative analysis **(C)** of pAMPK/AMPK (*n* = 6). **(D)** Docking analysis of NBP covalent binding mode to pAMPK. **(E, F)** NBP promoted pAMPK resistance to proteases by analyzing DARTS.

NBP has a small molecular weight, is highly fat-soluble, and easily passes through the blood-brain barrier. It is postulated that NBP has the capability to penetrate the cell membrane and directly interact with specific adaptor molecules. In order to identify the precise target of NBP, the structural data of SDF form of NBP was subjected to PharmMapper analysis using the “All Targets” model. The resulting list comprised the top 300 potential targets (job ID: 230131073910) (Supplementary Table S1), ranked in descending order based on the normalized fit score. Since mitochondrial fusion is normally regulated by kinases, functional annotations select the candidates that contain “Participate in kinase activity” and identify the five candidates, as shown in Figure 3A, which are “Proto-oncogene serine/threonine-protein kinase Pim-1” (Rank 2), “Mitogen-activated protein kinase 14” (Rank 25), “Serine/threonine-protein kinase Chk1” (Rank 40), “cAMP-dependent protein kinase catalytic subunit alpha” (Rank 55), and “Adenosine kinase” (Rank 58). According to the information provided by Universal Protein Resource (UniProt), Proto-oncogene serine/threonine-protein kinase Pim-1 is involved in cell survival and cell proliferation; Mitogen-activated protein kinase 14 is one of the four p38 MAPKs which play an important role in inflammatory response; Serine/threonine-protein kinase Chk1 regulates cell cycle progression; Arginine kinase catalyzes the reversible transfer of the terminal phosphoryl group of ATP to L-arginine. The biological functions of these four kinases are not related to the mitochondrial protective effects of NBP. Hence, AMPK was considered to be the most likely target candidates.

AMPK is a key direct regulator of mitochondrial dynamics. It can be activated by its upstream kinase or protect cells from pathological stimuli by responding to a decrease in ATP/AMP or ATP/ADP ratio. Hence, AMPK was identified as the primary candidate for targeting. Figures 3B, C also showed that NBP significantly increased pAMPK/AMPK protein expression in the MCAO/R-induced brain tissues. To further investigate the interaction between NBP and AMPK, computational docking was employed. The AutoDock Vina binding test revealed a potential favorable binding of NBP to pAMPK, with a maximum binding affinity of −5.7 kcal/mol (Figure 3D). To further support the direct interaction of NBP, a DARTS experiment was conducted using tissue lysates. The results from Figures 3E, F obtained from DARTS analysis demonstrated that the concentration of NBP exhibited inhibitory effects on p-AMPK degradation induced by pronase. These results suggest a direct interaction between NBP and AMPK.

### Inhibition of AMPK with dorsomorphin partly blunted the neuroprotective effects of NBP in MCAO/R-induced mice

**FIGURE 4:**
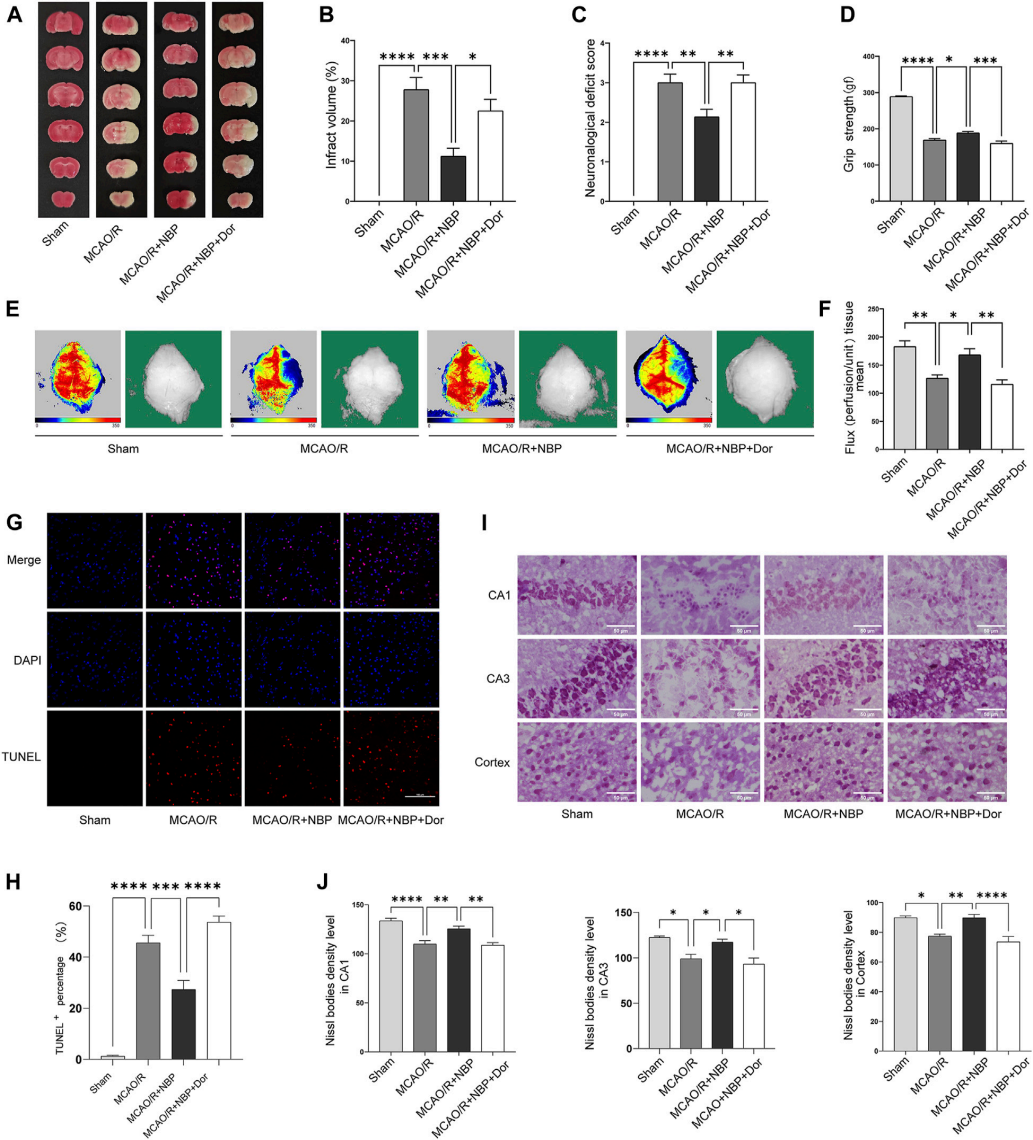
NBP alleviated infarction volume, neuronal loss, and facilitated restoration of cerebral blood flow, partly reversed by the inhibitor dorsomorphin. Mice were pretreated with or without NBP in the presence or absence of dorsomorphin for 7 d. **(A)** Effects of dorsomorphin on infarction volumes intervened with NBP. **(B)** Quantitative analysis of infarct volumes (*n* = 5). **(C)** Longa score (*n* = 12–16). **(D)** Grasping ability test (*n* = 11–15). **(E)** The images of cerebral blood flow of cortex. The magnitude of cerebral blood flow is represented by different colors, with blue to red indicating low to high. **(F)** Quantitative analysis of cerebral blood flow in different groups (*n* = 5–6). **(G)** TUNEL staining in the cortex region of each group (*n* = 3). **(H)** TUNEL^+^ percentage in the cortex region. Scale bar = 100 μm. **(I)** Nissl staining in the hippocampal and cortex regions of each group (*n* = 3–7). **(J)** Density levels of the Nissl staining in hippocampal CA1, CA3 and cortex regions. Scale bar = 50 μm. Data are expressed as the mean ± SEM and were analyzed by ANOVA, *p < 0.05, **p < 0.01, ***p < 0.001, and ****p < 0.0001.

To explore whether AMPK is involved in anti-cerebral I/R injury effect of NBP, AMPK inhibitors (dorsomorphin) was used to inhibit AMPK in MCAO/R-induced mice pretreatment with NBP. As shown in Figures 4A, B, the MCAO/R-induced cerebral infarct volume was suppressed by NBP, and this neuroprotective effect was weakened after blockade of the AMPK pathway. Moreover, pretreatment with NBP could relieve neurological dysfunction, increase grip strength and cerebral blood flow, inhibit cell apoptosis, improve Nissl’s body morphology after I/R injury in mice, but this effect was also blocked following treatment with dorsomorphin (Figures 4C–J).

### Inhibition of AMPK with dorsomorphin partly blunted the expression on Mfn1 of NBP in MCAO/R-induced mice

**FIGURE 5:**
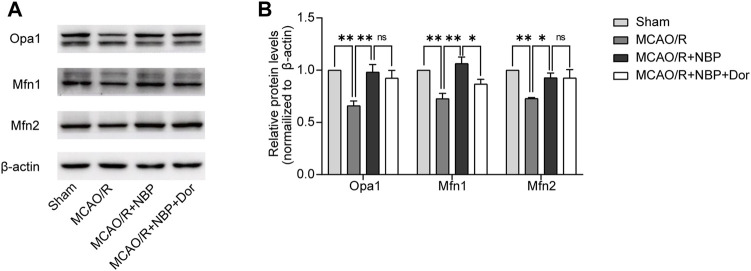
NBP promoted mitochondrial fusion and restored mitochondrial function, partly reversed by the inhibitor dorsomorphin. Mice were pretreated with or without NBP in the presence or absence of dorsomorphin for 7 d. **(A, B)** Representative blots and quantitative analysis of mitochondrial fusion-related proteins including Opa1, Mfn1, and Mfn2 (*n* = 3). Data are expressed as the mean ± SEM and were analyzed by ANOVA, *p < 0.05 and **p < 0.01.

To determine whether NBP regulates mitochondrial fusion via the AMPK pathway, we continue to use dorsomorphin to inhibit AMPK in MCAO/R-induced mice pretreatment with NBP. We analyzed the expression patterns of mitochondrial fusion proteins. Of the expression of Opa1, Mfn1 and Mfn2, only the expression of Mfn1 was significantly lower in the dorsomorphin and NBP co-treated group than in the NBP-treated group. No significant differences in Opa1 and Mfn2 level were observed in the dorsomorphin and NBP co-treated group, which suggests that Mfn1 expression were modulated by the AMPK signaling pathway (Figures 5A, B).

## Discussion

NBP, a compound derived from celery seeds, received approval from the National Medical Products Administration in 2002 for clinical treatment of ischemic stroke (Yao et al., 2018). The protective mechanism of NBP against ischemic stroke may be related to improving local microcirculation, inhibiting neuronal apoptosis and neuroinflammation (Wang Y. et al., 2018; Wei et al., 2021). However, further research is needed on the related studies of NBP on mitochondrial dynamics and its mechanism of action in brain I/R injury. We investigated the effect of NBP on MCAO/R-induced ischemic stroke and its potential mechanism involving mitochondrial fission. Our results showed that NBP reduces cerebral infarction volume, improves neurological dysfunction, and increases cerebral blood flow. NBP also increased the protein expression of mitochondrial fusion proteins Opa1, Mfn1 and Mfn2, and mitochondrial respiratory chain complex IV. In addition, to verify the relationship between AMPK and mitochondrial fusion, we injected the AMPK inhibitor dorsomorphin intraperitoneally and examined the expression of these fusion proteins. The results showed that dorsomorphin could significantly inhibit the expression of mitochondrial fusion protein Mfn1 intervened with NBP. These results suggest that NBP may promote mitochondrial fusion and ultimately alleviate I/R symptoms by regulating the AMPK-mediated Mfn1 pathway.

Mitochondria are present in most cell types and are the main productive structure. Mitochondria contain many enzyme systems, which can be coupled with oxidative phosphorylation through electron transport chains to generate adenosine triphosphate, providing huge energy for life activities (Annesley and Fisher, 2019; Qin et al., 2021; Al Amir Dache and Thierry, 2023). The realization of mitochondrial function is inseparable from structural changes. Mitochondrial fusion and division are fundamental to function. In typical scenarios, the processes of mitochondrial fusion and division operate in harmony, ensuring a dynamic equilibrium that upholds the cellular morphology, structure, and functionality of mitochondria (Youle and van der Bliek, 2012; Adebayo et al., 2021).

Fusion is an intracellular adaptation that enhances communication between mitochondria and the host cell (Bertholet et al., 2016). Developing therapeutic strategies to regulate mitochondrial fusion process is of utmost importance. A mounting body of empirical evidence highlights the significance of various fusion proteins, such as Opa1, and Mfn1/2. Therefore, there is a critical need to develop a novel drug target that regulates this crucial biological process. The data presented in this study indicate that NBP administration leads to an increase in the protein expression of Opa1, Mfn1, and Mfn2 in brain tissues affected by MCAO/R. Furthermore, the mitochondrial respiratory chain complex plays a crucial role in biological oxidation processes, primarily by facilitating the transport of electrons and protons to generate ATP (Rich and Maréchal, 2010). Complex IV, as the terminal complex of the respiratory chain, utilizes the proton gradient to synthesize ATP, thereby enabling energy conversion (Rich and Maréchal, 2010; Kadenbach, 2021). NBP has demonstrated efficacy in restoring the expression of mitochondrial complex IV in brain tissues subjected to MCAO/R. Taken together, these data indicated that NBP promoted mitochondrial fusion and mitochondrial function in I/R brains.

AMP-activated protein kinase (AMPK) is a crucial serine-threonine (Ser/Thr) protein kinase complex involved in the regulation of cellular energy status and monitoring of cellular metabolic state. Its activity is primarily modulated by the AMP/ATP ratio, and its activation can also be facilitated by its upstream kinase. Under conditions of stress, AMPK initiates the process of biosynthesis to generate new mitochondria as a means of replacing damaged ones (Toyama et al., 2016). Upon activation, AMPK is capable of directly phosphorylating serine or threonine residues on target molecules (Li et al., 2016) thereby regulating mitochondrial fission through its control over the mitochondrial fission factor, a key receptor on the outer membrane for Drp1 (Toyama et al., 2016). In this study, we have observed that NBP activates AMPK. Furthermore, the inhibitory effect of NBP on Mfn1 at the mitochondrial membrane is dependent on the presence of AMPK, as demonstrated through AMPK silencing experiments. These findings indicate that the protective impact of NBP on IS is, in part, achieved by activating the AMPK-mediated Mfn1 signaling pathway.

## Conclusion

Our research findings indicate that NBP has the ability to modulate mitochondrial homeostasis by activating AMPK, leading to the mitigation of cerebral I/R injury. Importantly, our study presents novel evidence that the administration of NBP can effectively decrease infarct volume and enhance neurological functions by facilitating AMPK-mediated mitochondrial fusion in *in vivo* models of ischemic stroke.

## Data Availability

The original contributions presented in the study are included in the article/Supplementary Material, further inquiries can be directed to the corresponding author.
