# Supplementary figures and images for: Dl-3-n-butylphthalide attenuates cerebral ischemia/reperfusion injury in mice through AMPK-mediated mitochondrial fusion
Source: Front Pharmacol. 2024 Feb 22;15:1357953. doi: 10.3389/fphar.2024.1357953 (PMC10917971; doi:10.3389/fphar.2024.1357953)

**
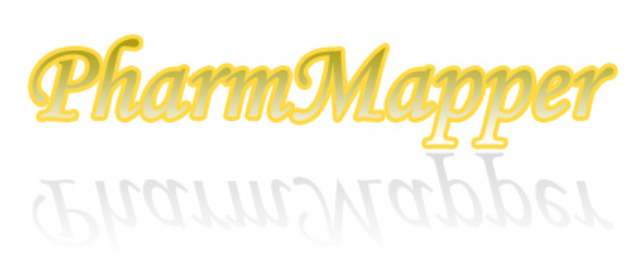
Table S1**


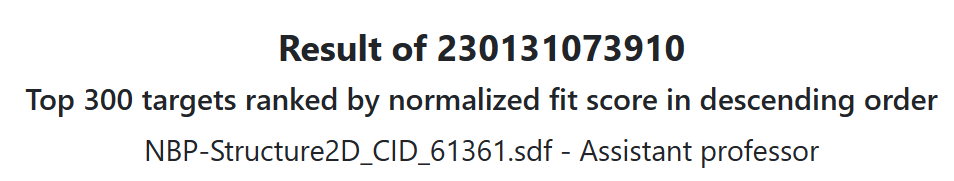


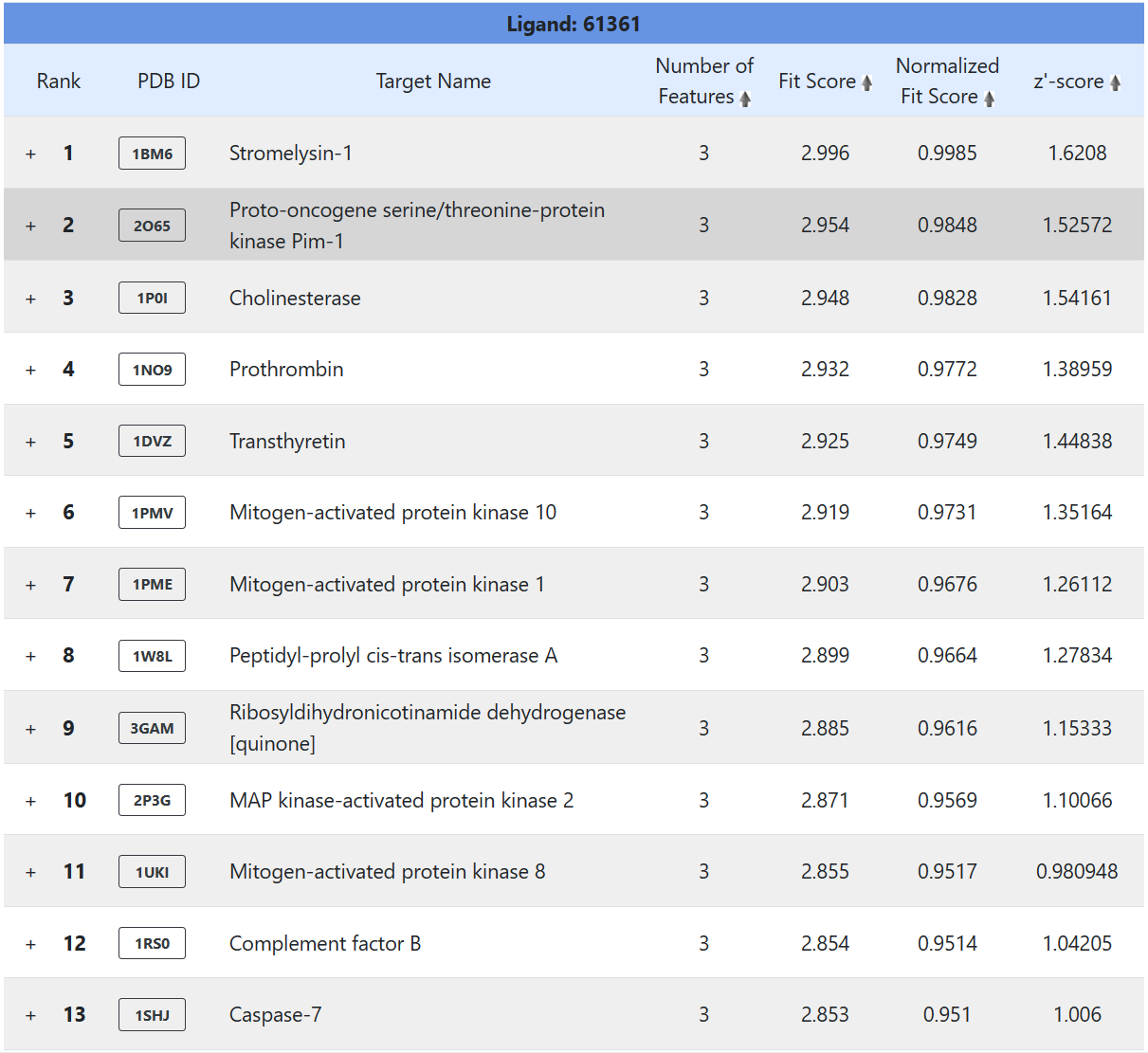


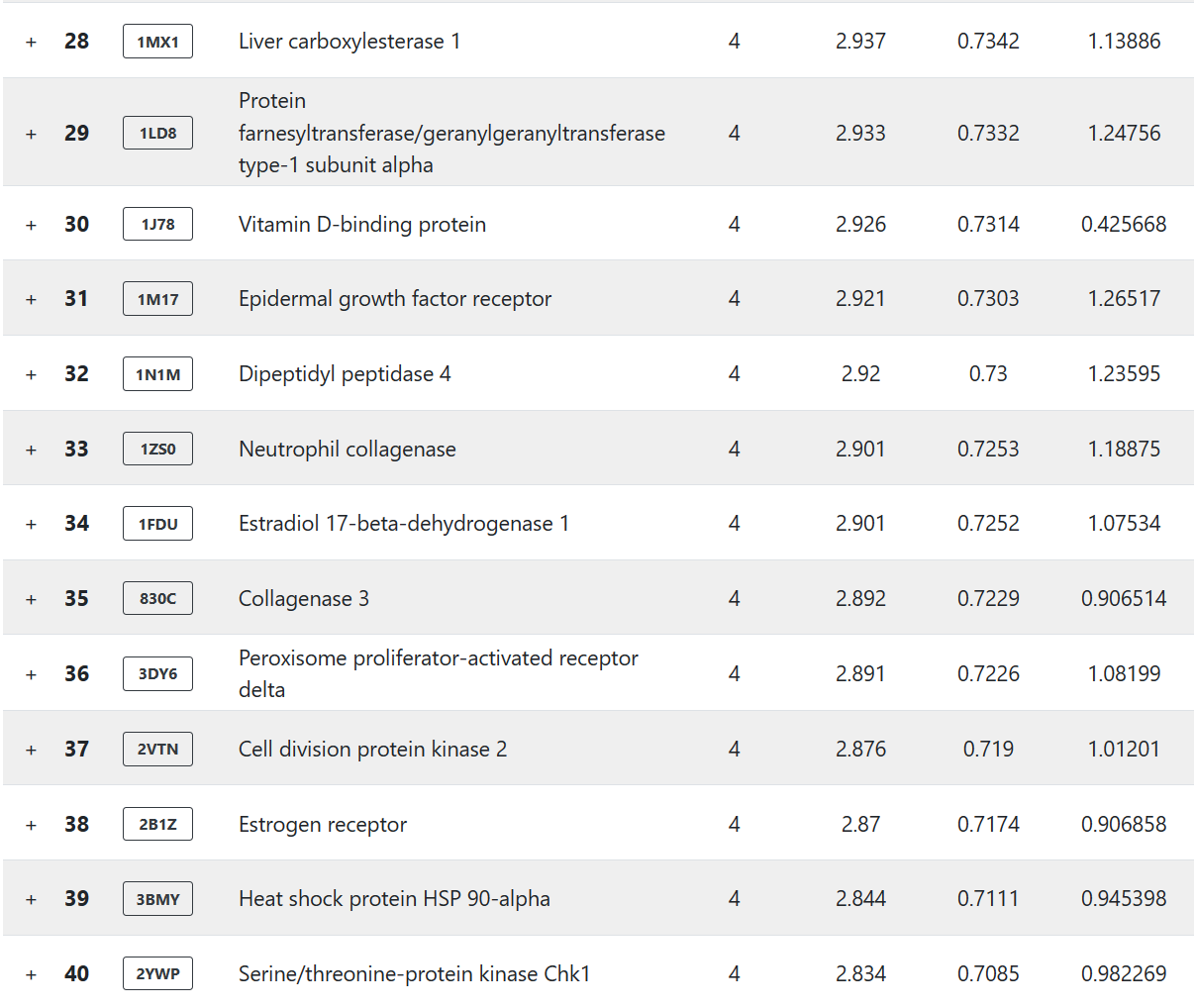

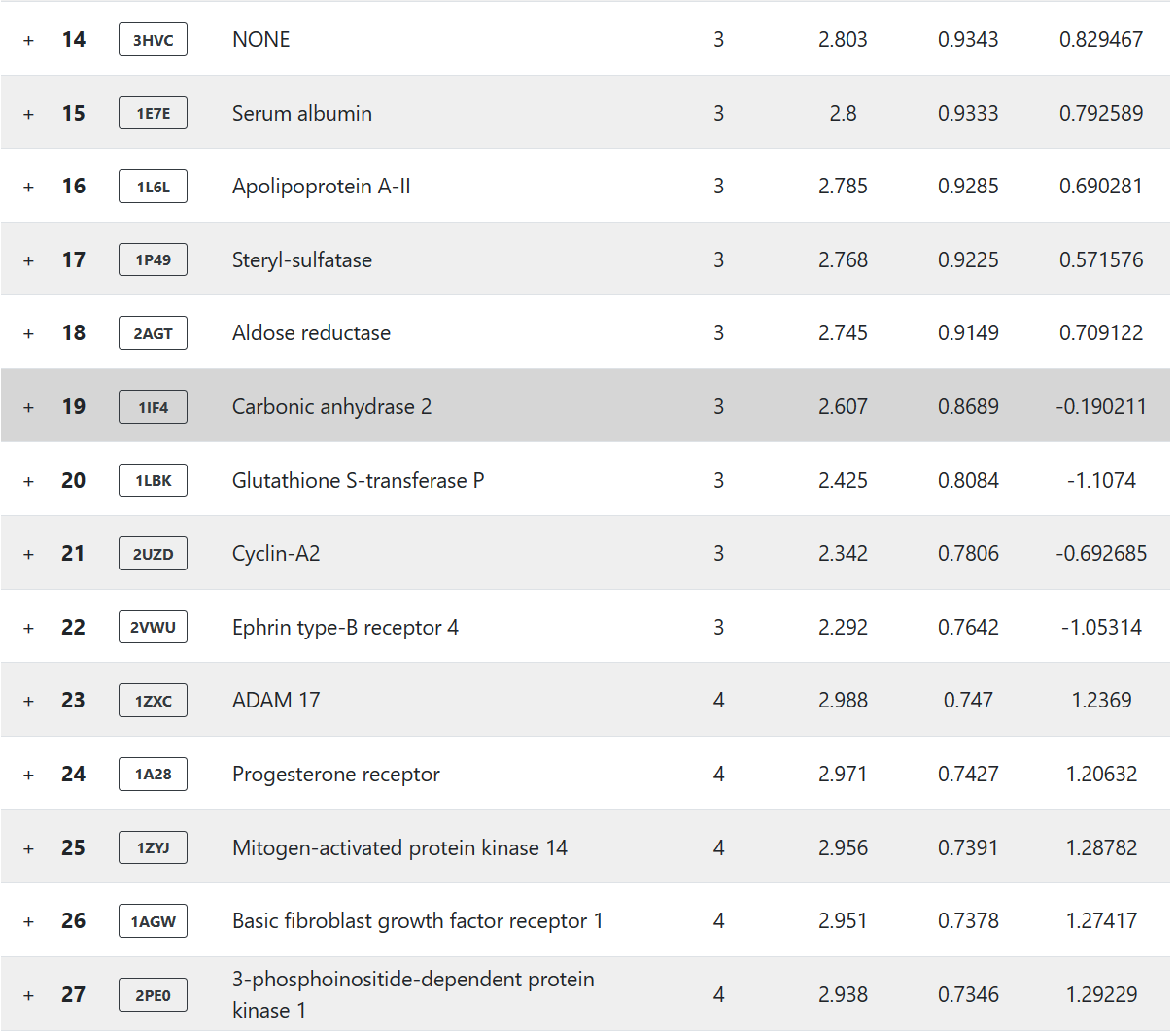

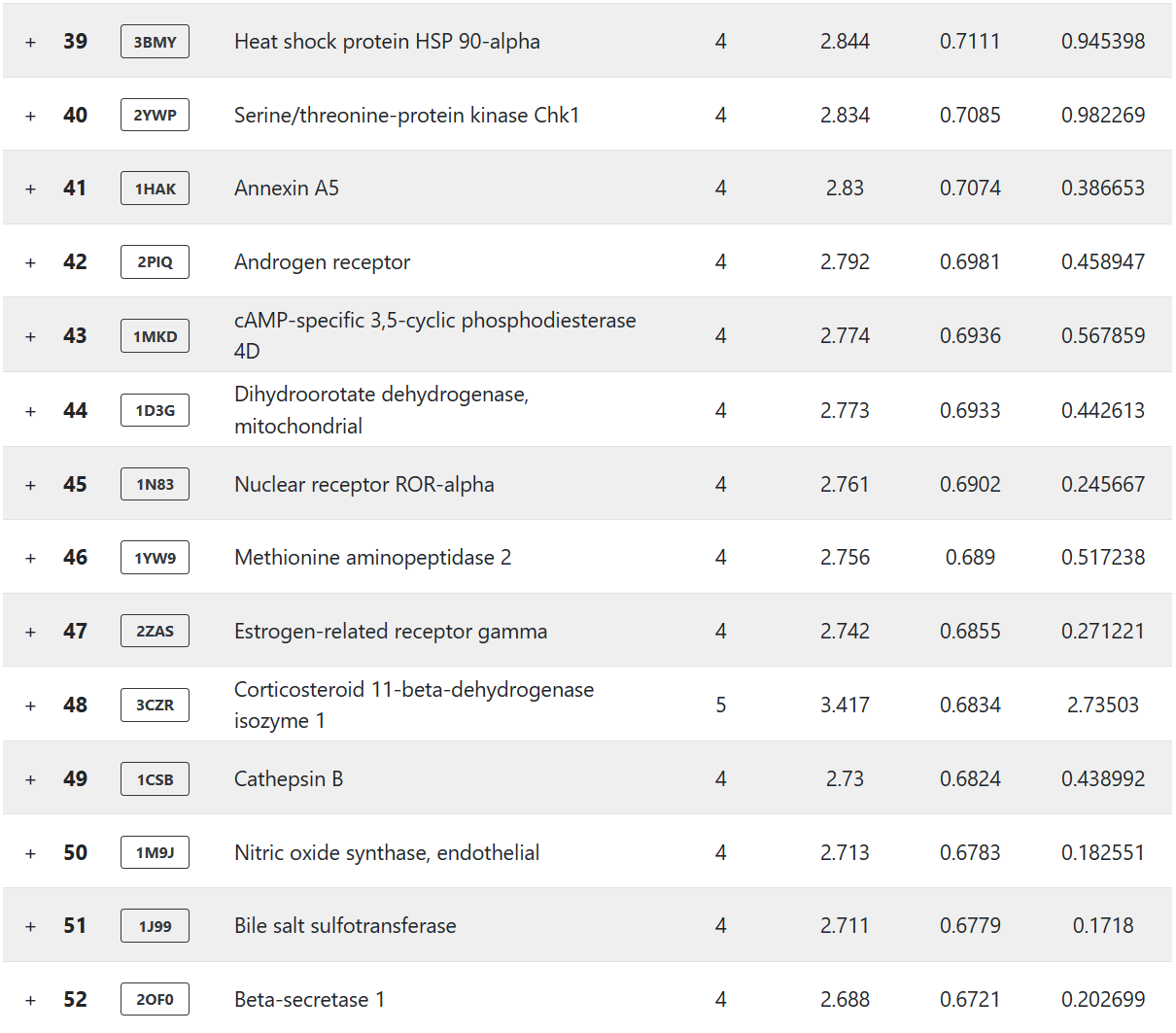

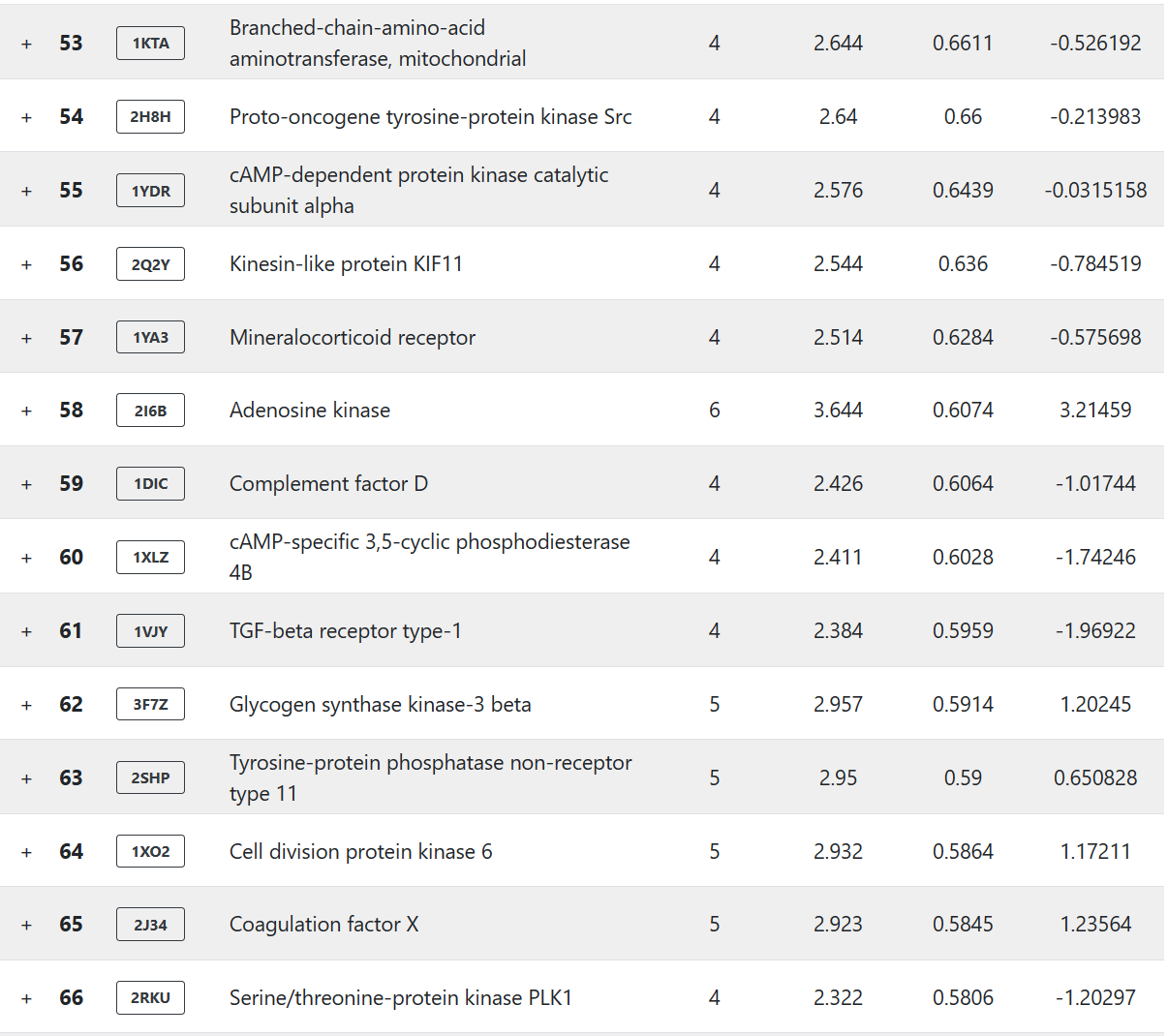

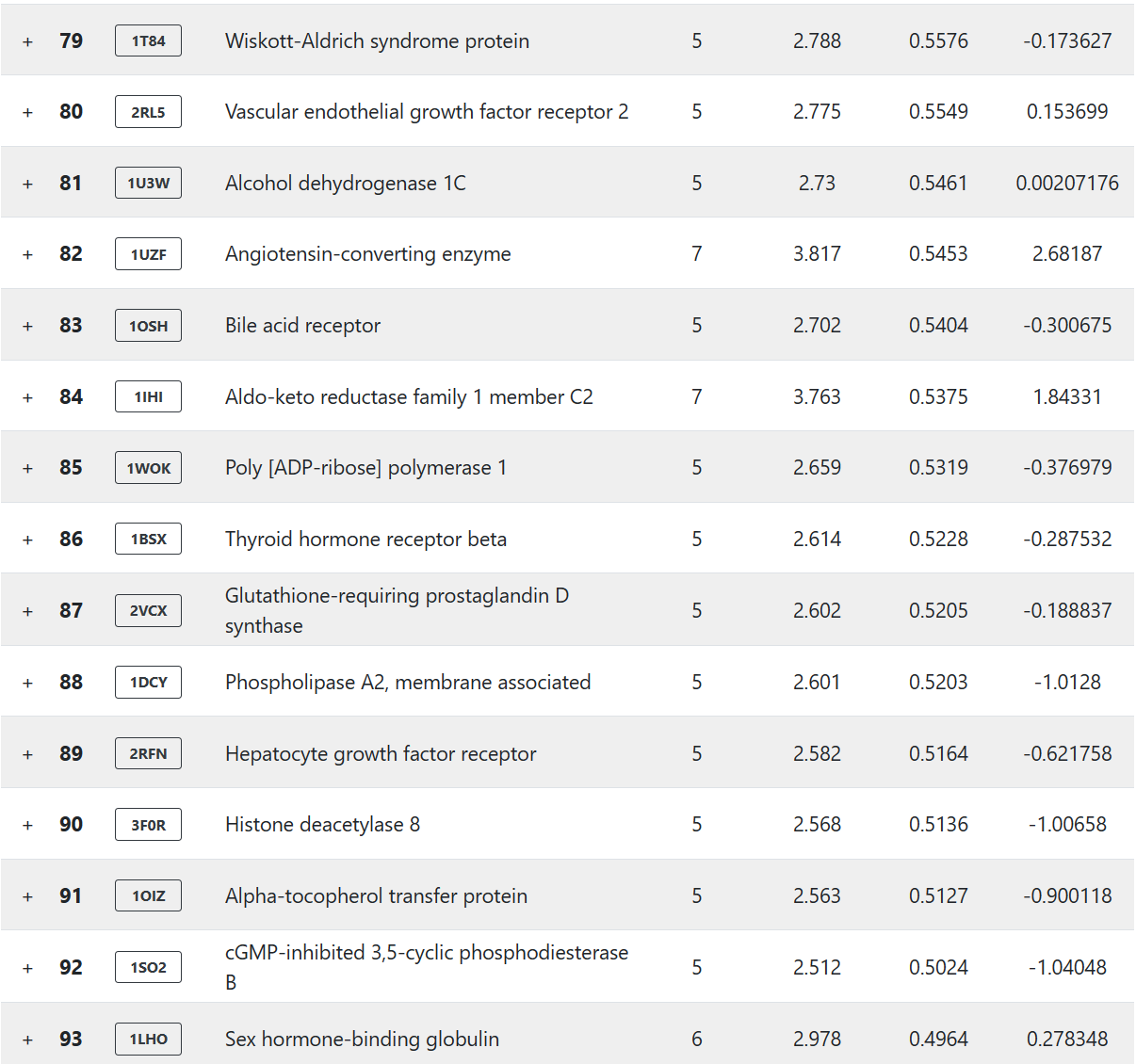

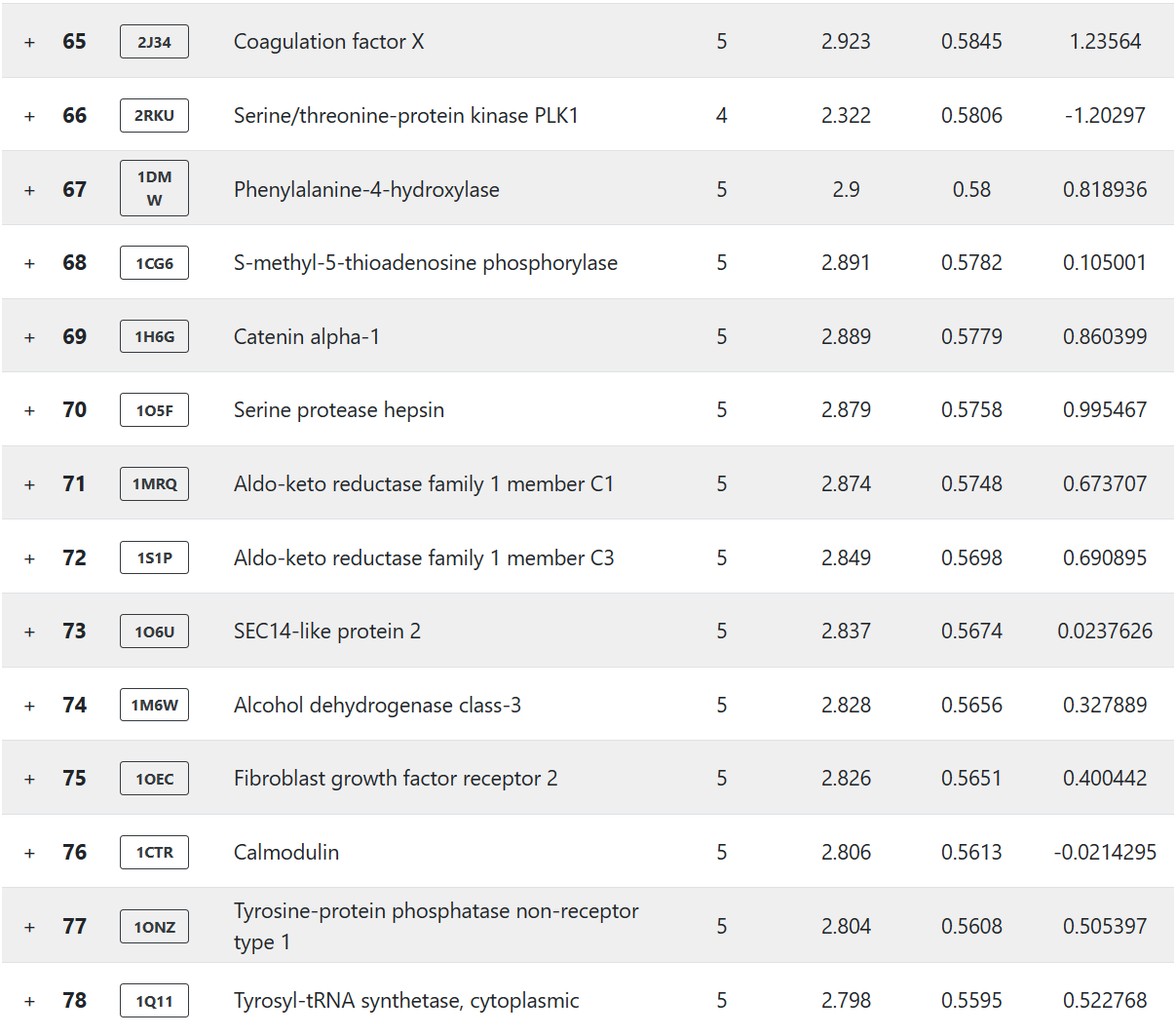

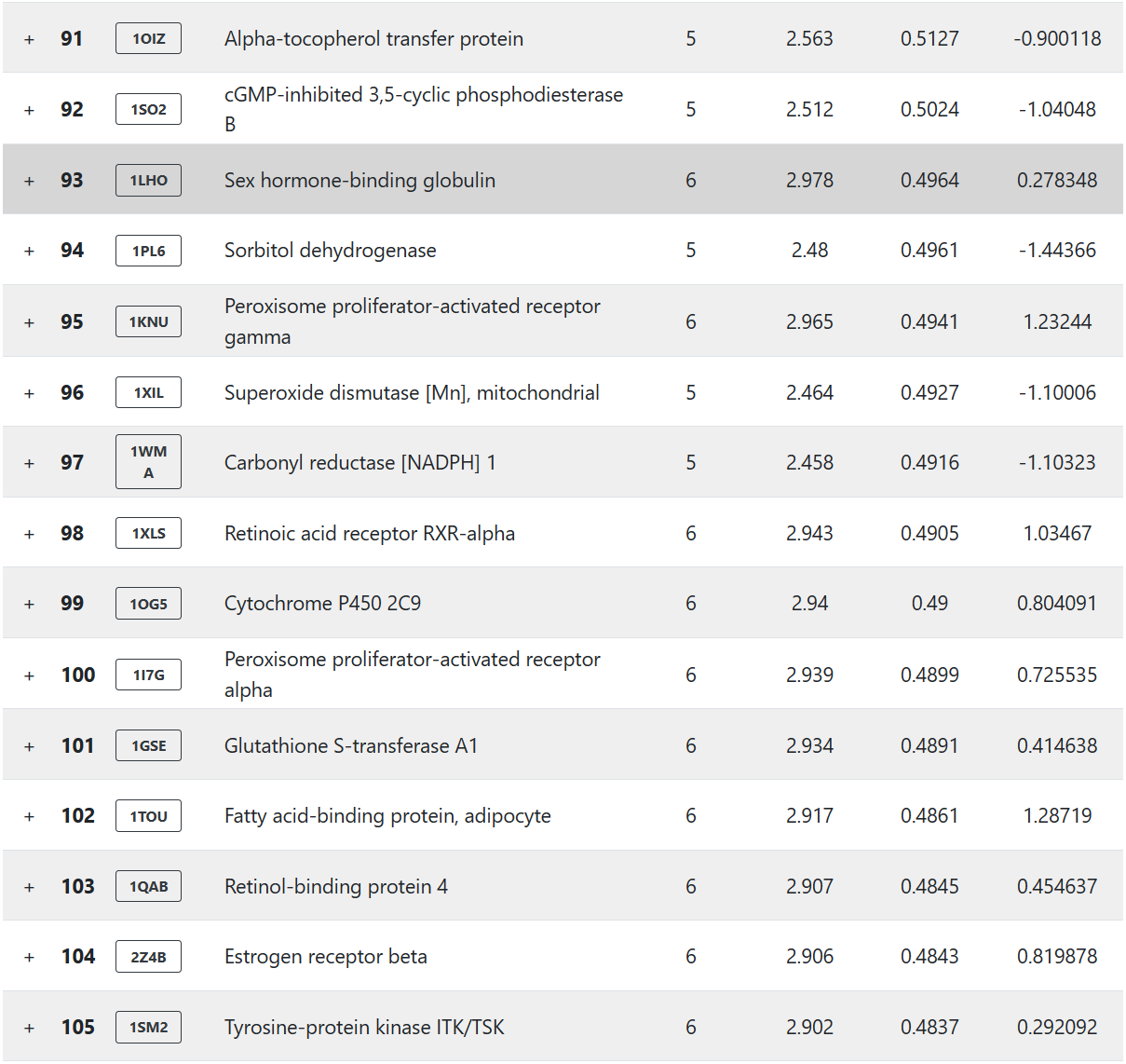

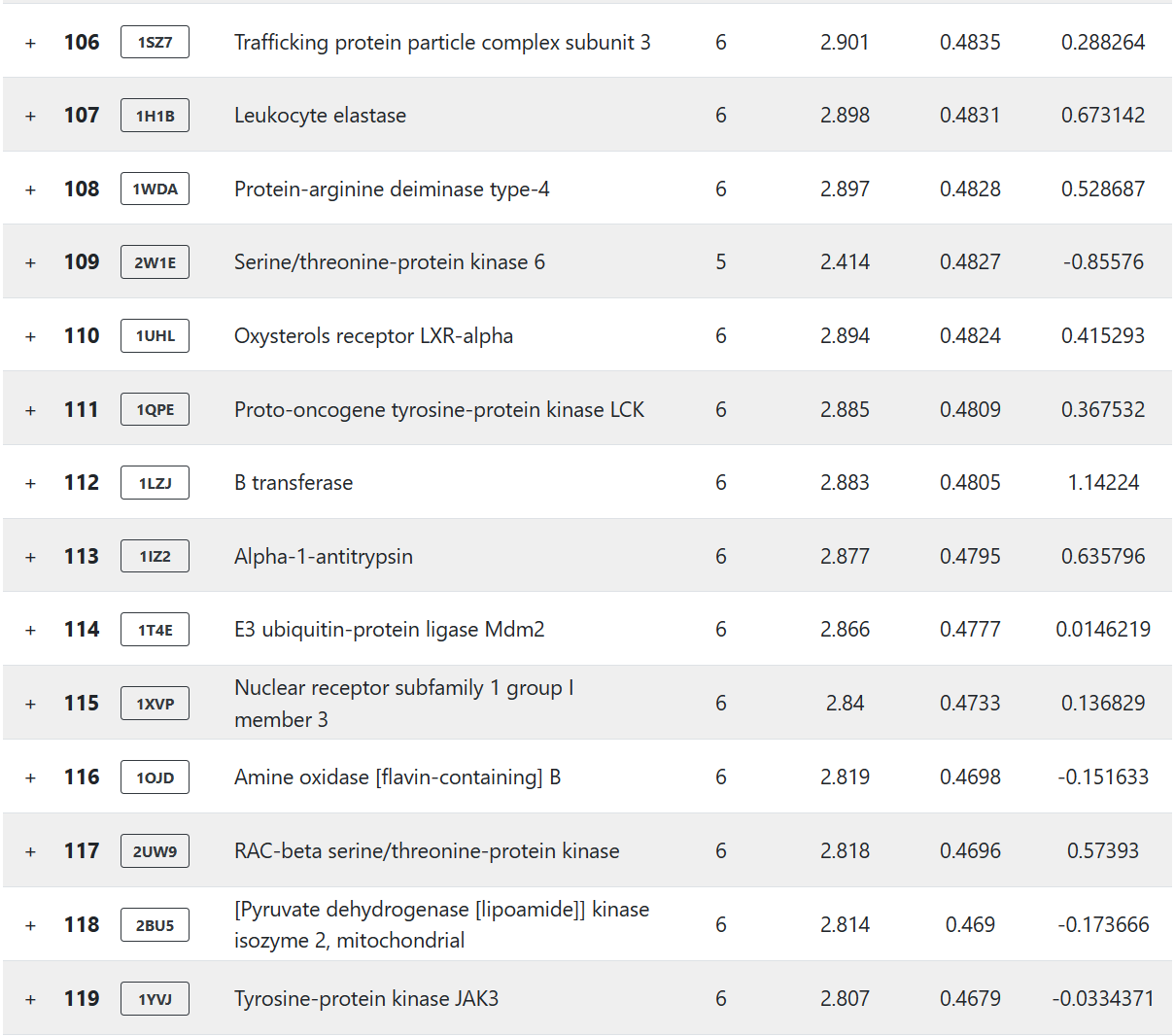

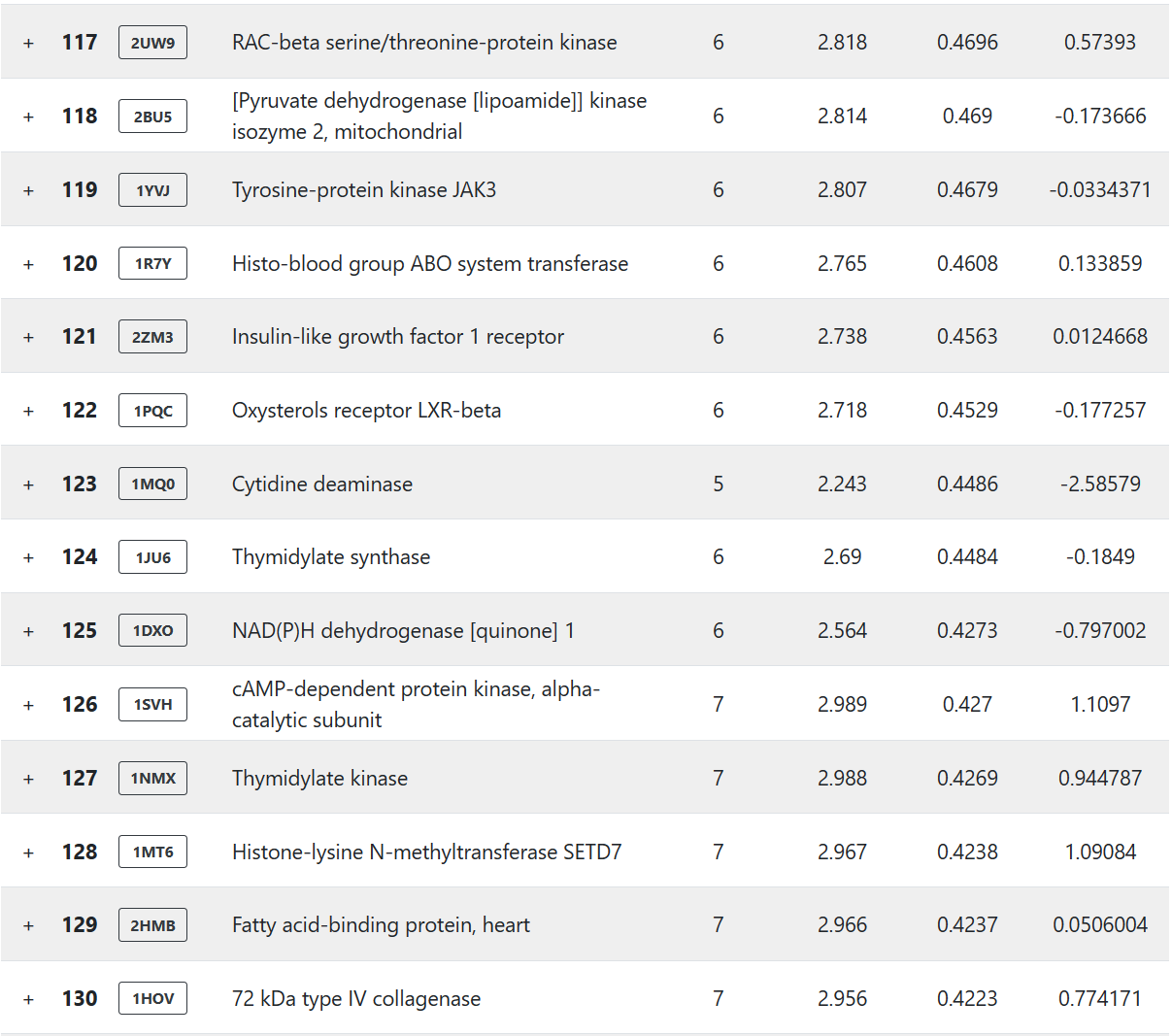

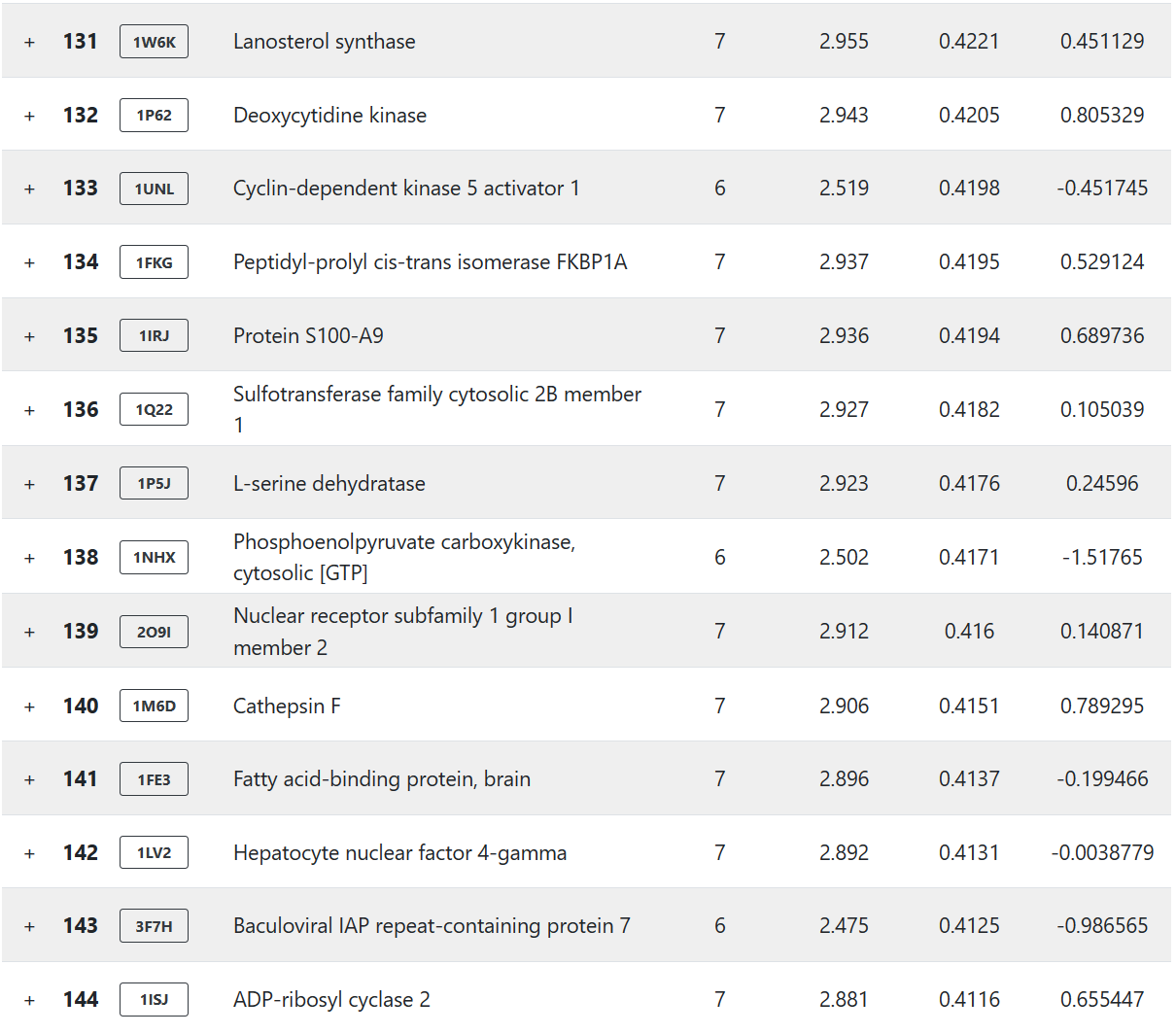

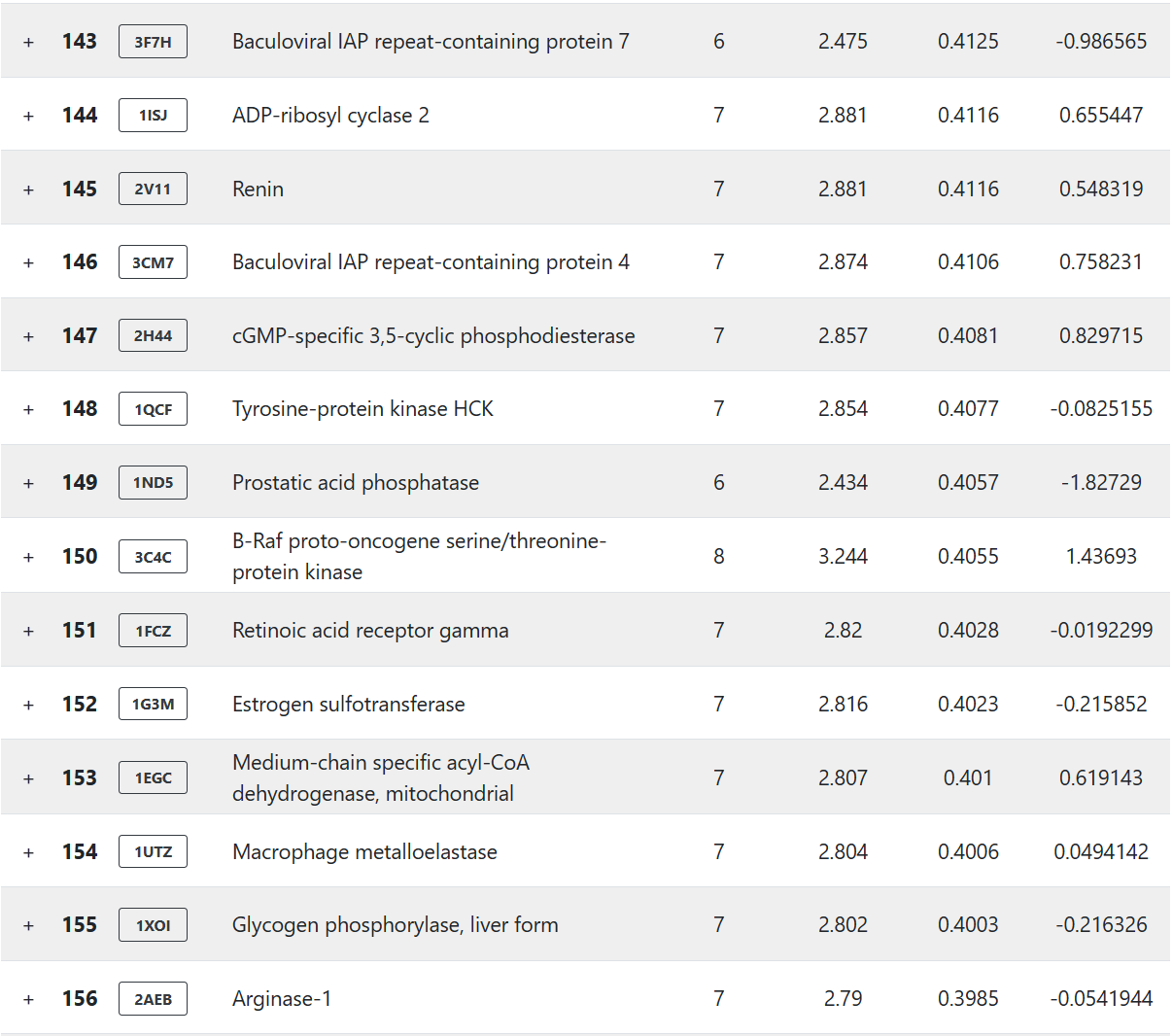

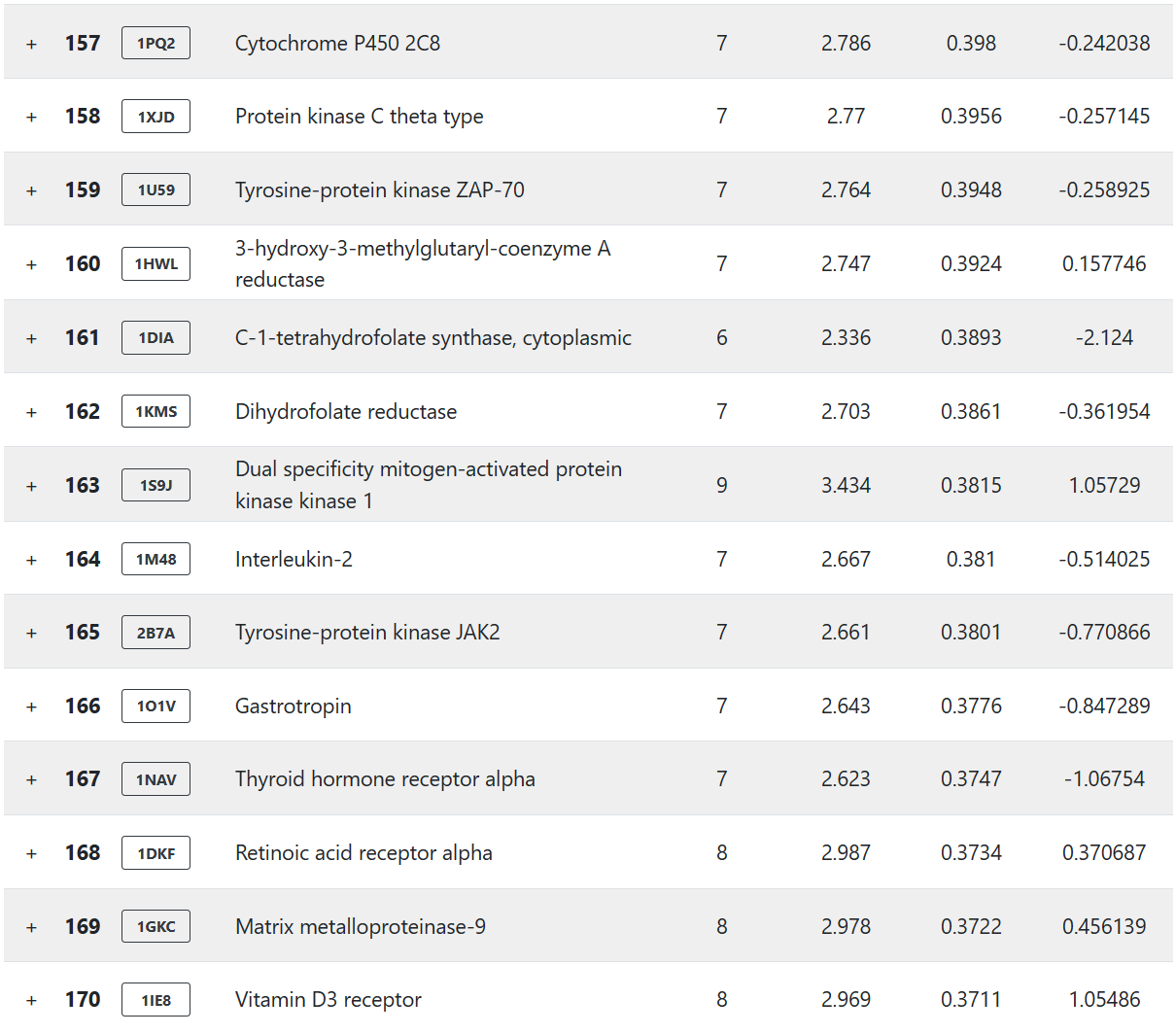

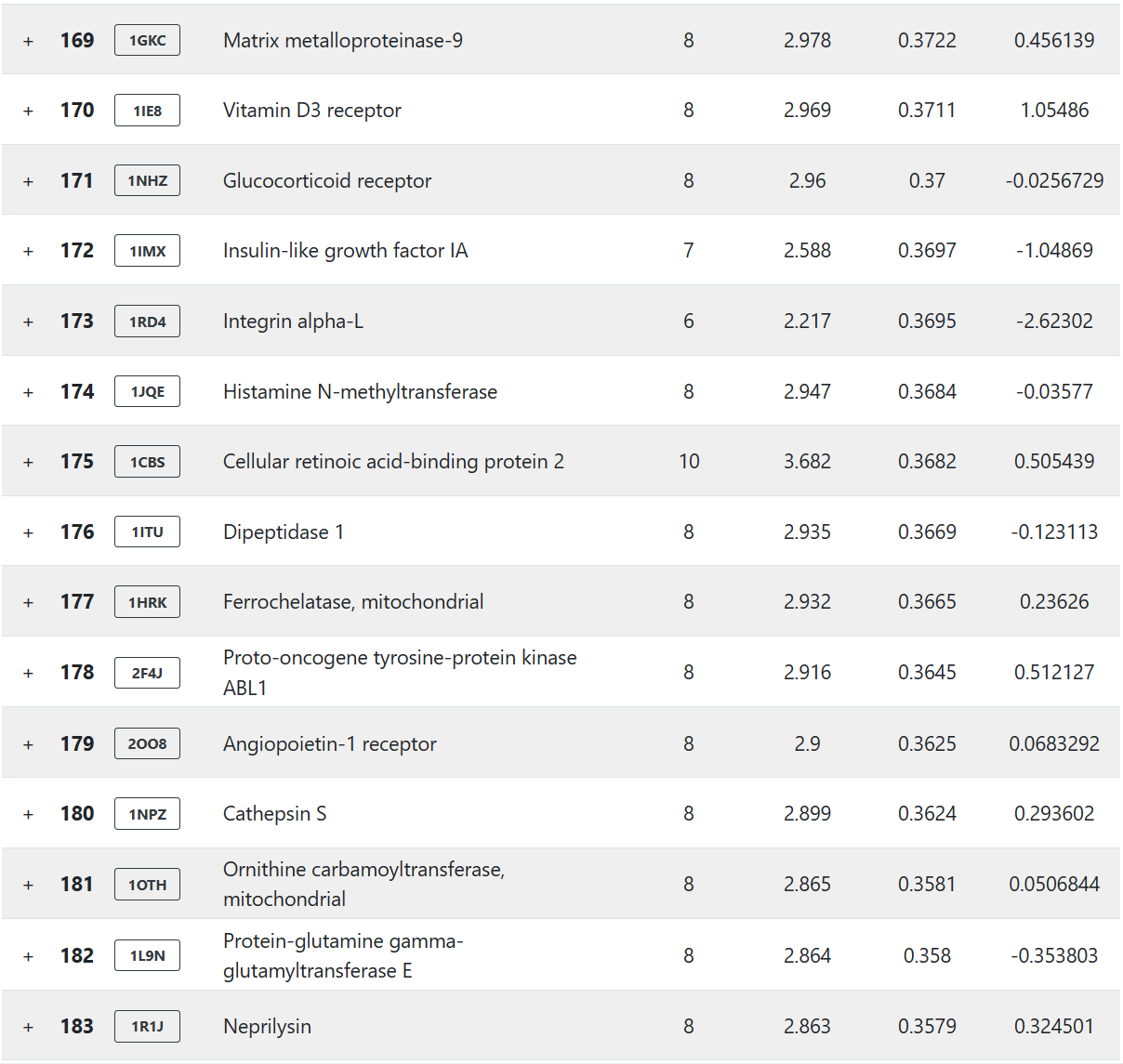

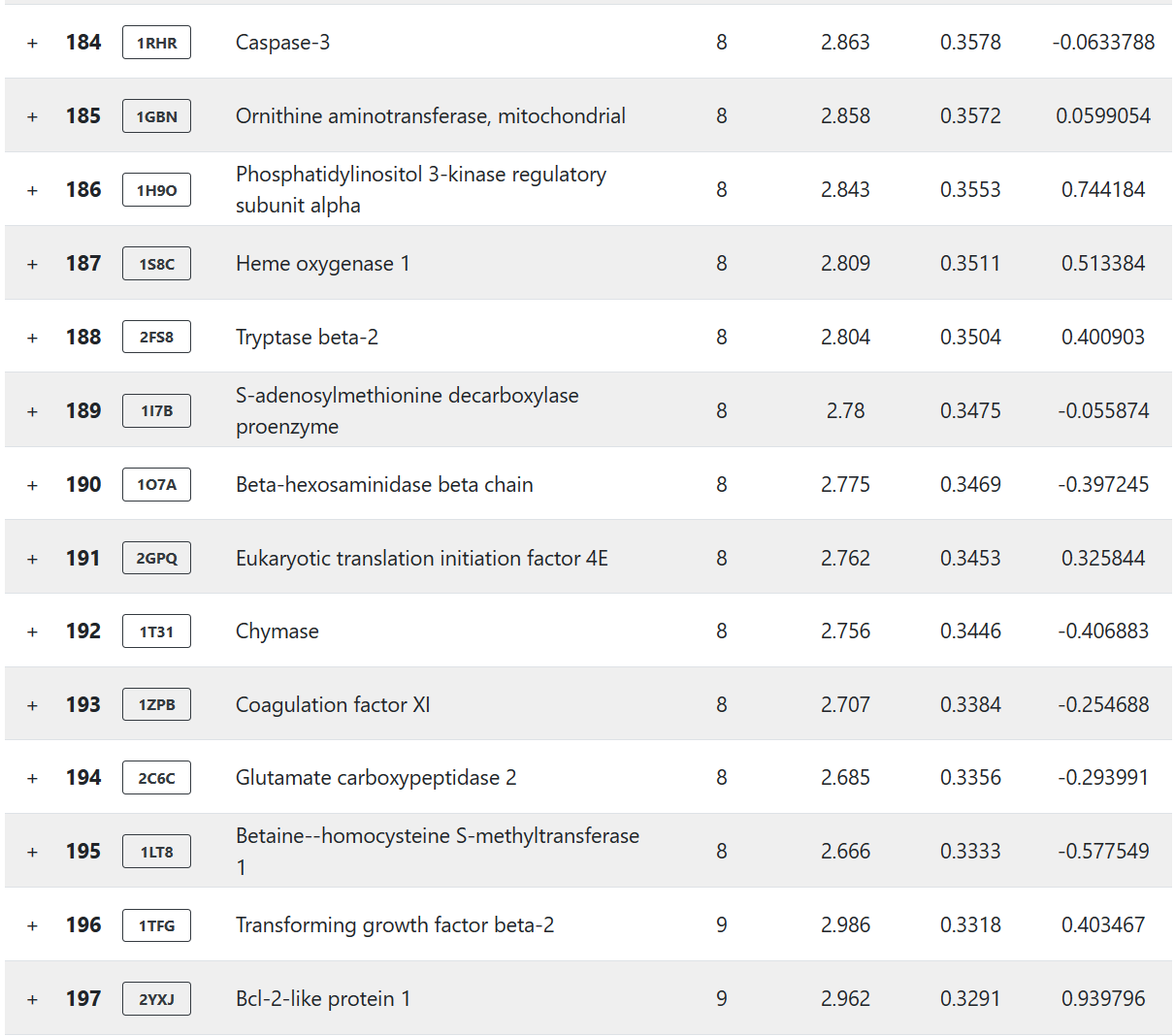

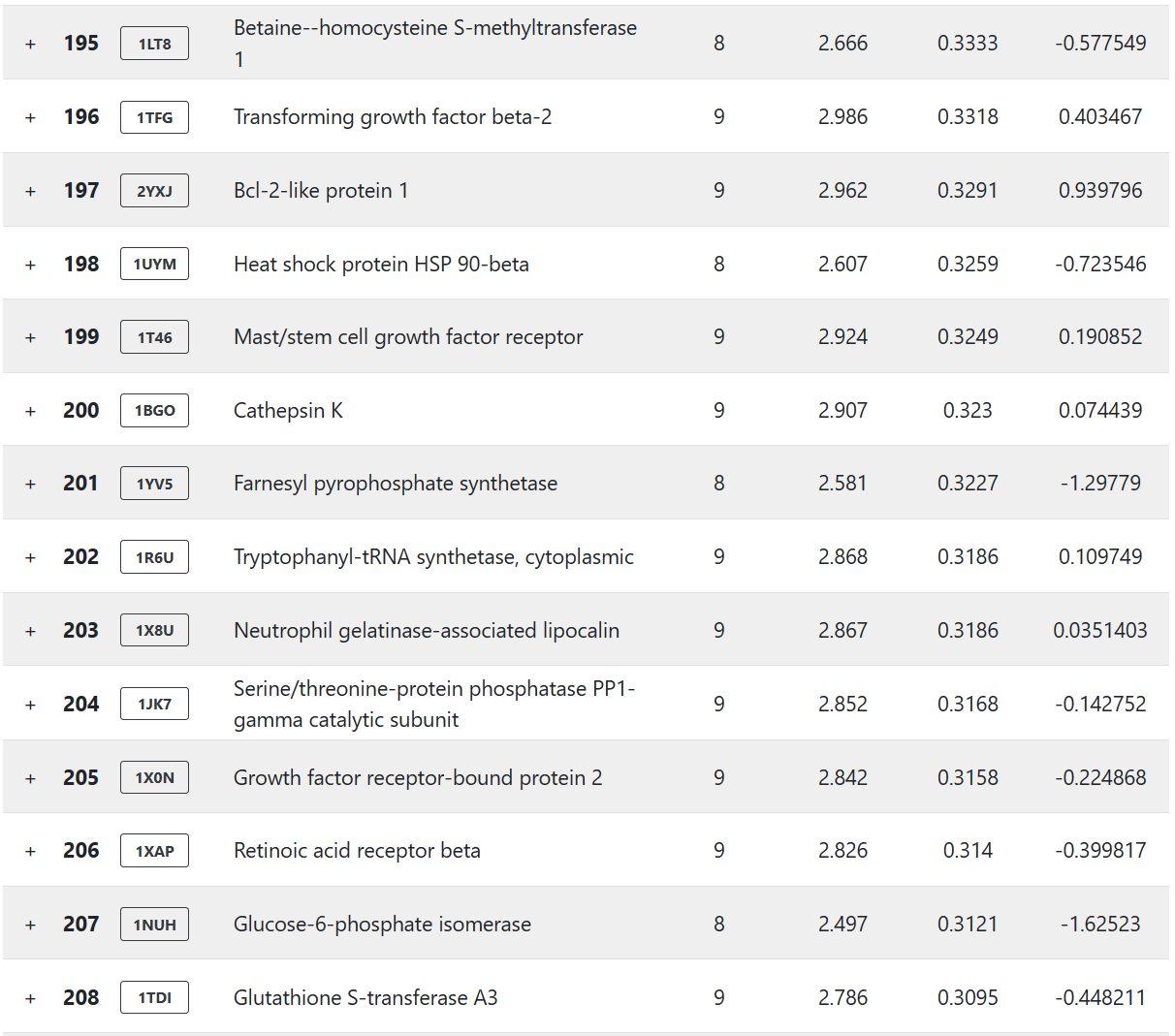

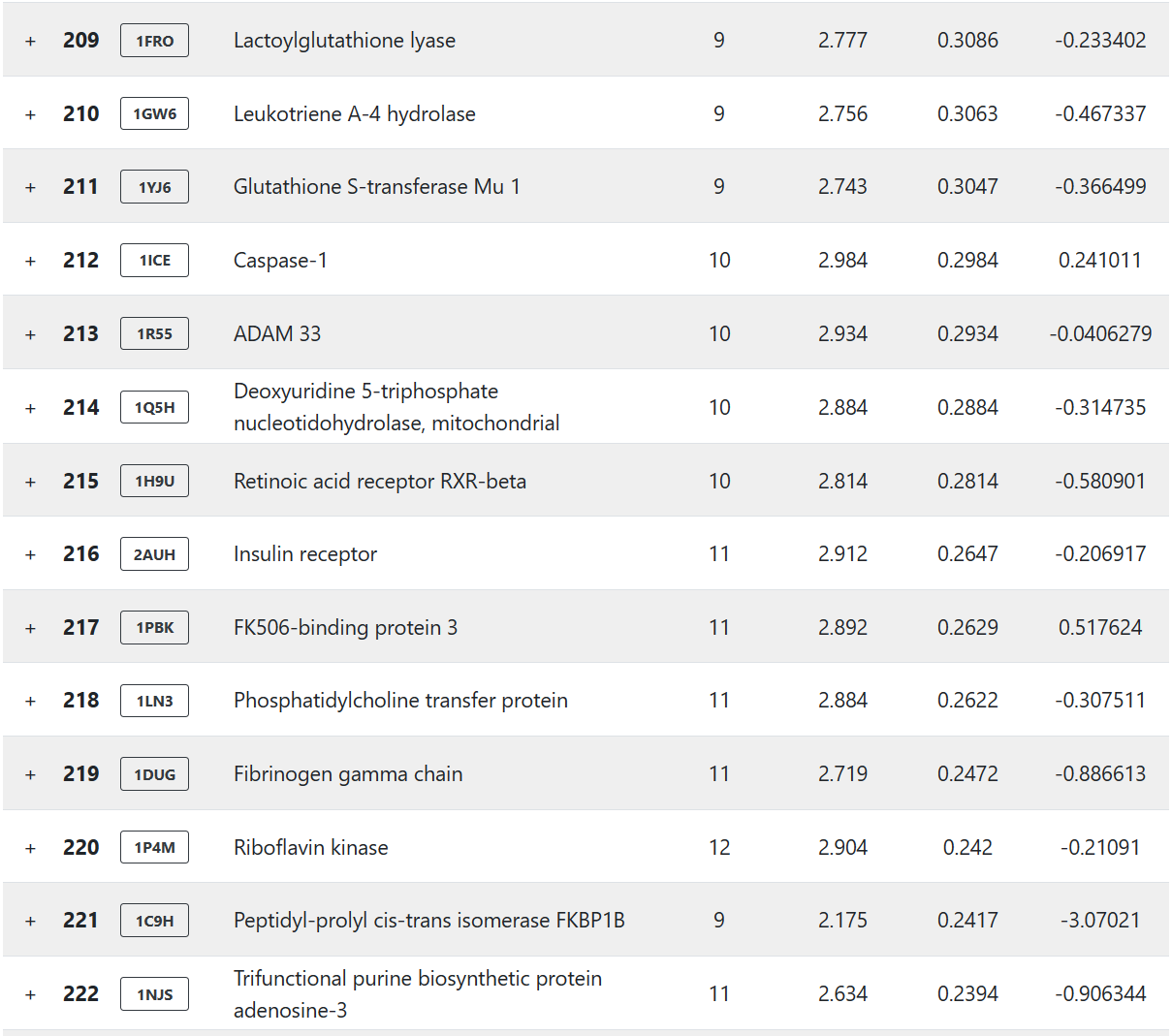


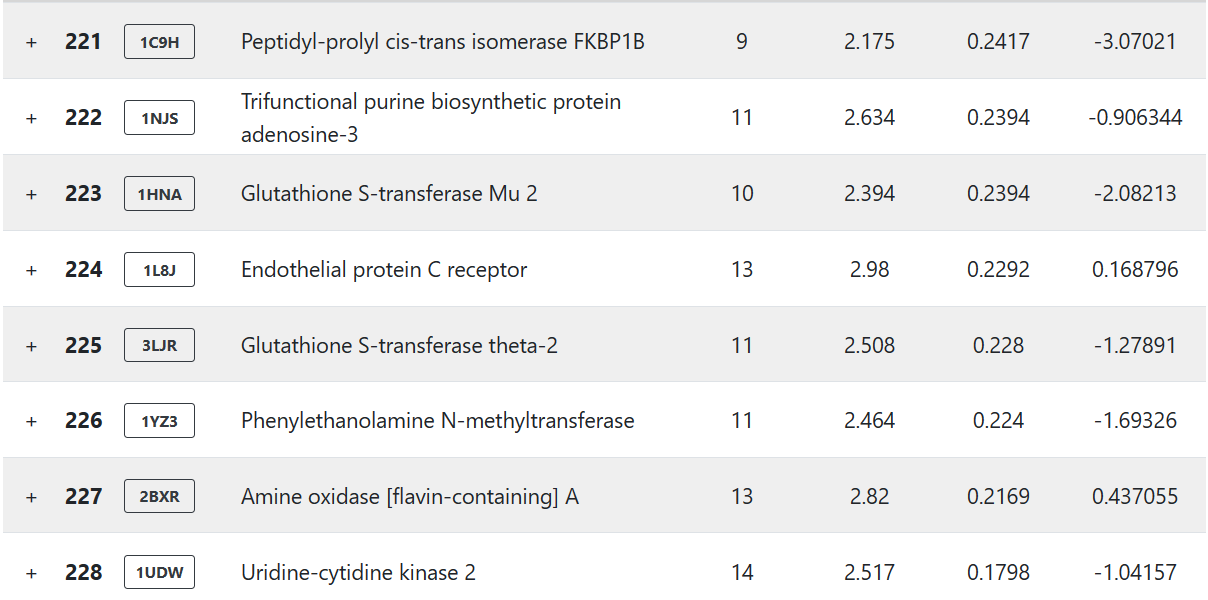

Supplement: Supplementary file 1 [file Table1.DOCX]
